# The comparative effectiveness of telehealth versus primary care and collection of urine cultures on outcome in urinary tract infection

**DOI:** 10.1097/MD.0000000000043172

**Published:** 2025-07-18

**Authors:** Karl J. Madaras-Kelly, Jeremy K. Boyd, Laura Bond

**Affiliations:** a College of Pharmacy, Idaho State University, Meridian, ID; b Boise VA Medical Center, Boise, ID; c Department of Biology, Boise State University, Boise, ID.

**Keywords:** comparative effectiveness, microbial culture, telehealth, urinary tract infection

## Abstract

Studies of urinary tract infection (UTI) treatment in telehealth settings have primarily evaluated young, healthy females. Urine culture collection is less common in telehealth settings but is recommended for all patients with potentially complicated infection. The aims of this study were: (1) to compare UTI-related clinical failure between telehealth and in-person primary care settings, and (2) evaluate if urine culture (UC) collection impacted UTI-related failure in patients with risk for antibiotic resistant infection. A retrospective cohort study of outpatients diagnosed with UTI between 2019 and 2021 in the Department of Veterans Affairs system was conducted. Inclusion required a telehealth or primary care visit with UTI International Classification of Diseases-Clinical Modification 10th revision code documentation and an antibiotic dispensed. Patients with recent UTI, concurrent indications for antibiotics, or where asymptomatic bacteriuria treatment was appropriate were excluded. Treatment failure was defined as combination of a new UTI-related outpatient visit or hospitalization that occurred between 3 to 30 days after the antibiotic dispense date. Antibiotic exposure data, covariates, hospitalization, and UC history were obtained. Overlap weighting and generalized estimating equation models estimated the relative risk of failure for clinical setting and for UC collection versus no collection. There were 16,266 telehealth and 29,296 primary care patient-visits evaluated. The adjusted relative risk (±95% CI) of failure for telehealth relative to primary care was [0.87 (0.70, 1.08)]. An interaction between setting and age ≥65 [1.45 (1.12, 1.87)] indicated higher failure for elderly patients treated for UTI in telehealth. Urine culture collection was associated with increased risk of failure for patients treated in telehealth [2.06 (1.56, 2.72)]; however, an interaction between UC collection and prior antibiotic exposure ≤90 days indicated a protective effect in both telehealth [0.70 (0.53, 0.93)] and primary care [0.77 (0.60, 0.99)] settings. Overall, no difference in the clinical failure rate for UTI treatment between telehealth and primary care was observed. However, elderly patients treated for UTI in telehealth experienced higher failure relative to in-person primary care. Patients with recent prior antibiotic exposure in both settings had lower clinical failure rates when UCs were collected.

## 1. Introduction

The United States Department of Health and Human Services established an objective for Healthy People 2030 to reduce urinary tract infection (UTI)-related hospitalizations for older adults.^[1]^ Treatment guidelines recommend that it is unnecessary to obtain a urine culture (UC) in young healthy females with lower UTI (i.e., cystitis) and empirical antibiotic treatment is sufficient.^[2,3]^ Infectious Diseases Society of America guidelines for the management of uncomplicated UTI recommend obtaining a UC if the patient was recently treated with antibiotics in cases of suspected pyelonephritis.^[2]^ Pending Infectious Diseases Society of America guidelines for complicated UTI suggest that UC should be obtained in all patients and that treatment selection be based on a history of colonization/infection with antibiotic-resistant bacteria or recent exposure to fluoroquinolones.^[4]^ Other treatment guidelines recommend obtaining UC in patients with a history of colonization or infection with antibiotic-resistant bacteria, recent antibiotic exposure or hospitalization, and in men with suspected UTI.^[5–7]^ All guidelines recommend collection of urinalysis (UA) and UC only for patients who have symptoms consistent with UTI unless pregnant or undergoing invasive procedures as asymptomatic bacteriuria is commonly overtreated.

Recently, diagnostic stewardship interventions including reflex culture policy revisions, order entry that requires testing justification, and audit-feedback that focus on limiting the collection of unnecessary UA/UC have reduced the treatment of asymptomatic bacteriuria.^[8,9]^ Clinicians frequently extrapolate diagnostic and treatment criteria from uncomplicated UTI to more complicated cases, irrespective of differences in patient populations and risk for clinical failure. Whether patients with risk factors for antibiotic-resistant bacteria benefit from UC collection is unknown.

Telehealth, video care, and asynchronous care, collectively defined as telehealth has increased substantially over the past decade, and UTIs are common infections treated in these settings.^[10–20]^ Potential advantages of telehealth include patient convenience, improved access for rural populations, and reduced health system cost. In some instances, telehealth specific triage and decision-making software that limits who the clinician can treat remotely and which diagnostic tests can be ordered contributes to cost savings.^[10,12]^ Potential telehealth disadvantages include lack of patient digital literacy and access, limited physical and/or laboratory assessment potentially resulting in misdiagnosis, and miscommunication of treatment recommendations between patient and clinician. Studies comparing the treatment of UTI in telehealth and traditional care settings have generally demonstrated higher prescription of guideline recommended antibiotics, equivalent or slightly lower return visit rates, and lower ordering of diagnostic tests including UA and UC in telehealth settings.^[12–17]^ Almost all of these studies limited the study population to women with uncomplicated UTI with limited comorbidity or risk for antibiotic-resistant bacteria.

During the coronavirus disease of 2019 (COVID-19) pandemic, care delivered by telehealth expanded exponentially including visits for nontraditional patient populations served by telehealth.^[10,11,21]^ This expansion included many healthcare systems with limited telehealth experience or standardized protocols for UTI triage, diagnosis, and treatment. While the Department of Veterans Affairs (VA) has delivered care via telehealth for more than a decade using specialized telehealth clinics, traditional in-person care settings now offer telehealth visits. Determining which patients should receive care through telehealth or how UTIs should be diagnosed and treated during telehealth visits is subject to local or regional VA control. Further, the Veteran population is slightly older, male, rural, and has more physical health disorders on average than the non-Veteran population.^[22]^ As both uncomplicated and complicated UTI are common diagnoses with potential for treatment via telehealth the objectives of this study were: (1) to compare UTI-related revisit rates between patients treated in telehealth and in-person primary care settings, and (2) to evaluate whether collection of UC impacted the UTI-revisit rates in patients with and without antibiotic resistance risk factors (i.e., risk for complicated infection) in these settings.

## 2. Methods

The study protocol [1681746-1] was reviewed by the VA Puget Sound Healthcare System, Seattle, WA. Institutional Review Board on May 19th, 2022, classified as minimal risk and “exempt” from further review per 2018 Common Rule Exemption 4iii (use of unidentifiable retrospective data). This research complies with all federal guidelines and VA policies relative to human subject’s research and followed the Strengthening the Reporting of Observational Studies in Epidemiology guideline and checklist.^[23]^

A national retrospective cross-sectional cohort study of outpatient Veterans diagnosed with UTI between January 1, 2019 and December 31, 2021 was conducted. Inclusion criteria required that patients received care through a Telehealth or Primary care setting, that an International Classification of Diseases-Clinical Modification 10th revision (ICD-CM 10) for UTI (see Supplement 1, Supplemental Digital Content, https://links.lww.com/MD/P435, inclusion & exclusion criteria and codes) was coded for the index visit, and that a prescription for an oral antibiotic of interest with a fill duration of <30 days was released to the patient on days 0 to 3 after the index visit.^[24]^ Antibiotics of interest included: β-lactams (amoxicillin, amoxicillin/clavulanate, cephalexin, cefuroxime, cefpodoxime, cefdinir), fluoroquinolones (levofloxacin, ciprofloxacin), nitrofurantoin, and trimethoprim/sulfamethoxazole . Fosfomycin was excluded due to sparse prescription for index visits. Other exclusion criteria were: UTI diagnosis within 30 days prior to index visit; hospitalization within 7 days prior to index visit; hospitalization or death within 2 days after index visit; prescription indicating treatment with antibiotics at the time of index visit; multiple antibiotics within 2 days of index visit; co-diagnosis of infection for which antibiotics may have been indicated; urological procedure 1 day before to 7 days after index visit; and test indicating pregnancy within 9 months prior to their index visit.^[25–27]^

Data were extracted from the VA Corporate Data Warehouse (CDW): patient demographics; visit date, time, and modality; vitals, laboratory values including UC & susceptibility results; chronic comorbidities; antibiotic resistance risk factor history (prior hospitalizations/skilled nursing facility residence, prior antibiotic exposures, antibiotic-resistant bacteria from prior cultures); duration of antibiotic prescribed; visit provider; practice setting; VA facility; and clinical outcomes of interest.^[28]^ UC results were classified by Gram stain morphology, genus, and antimicrobial susceptibility based on S/I/R reporting for antibiotic classes of interest for all reported organisms.

Telehealth visits were identified by review of primary and/or secondary clinic stop codes and their descriptions for visits with the terms “telehealth,” “telephone,” “video,” “vid,” or “secure messaging” in which all inclusion and no exclusion criteria were met. Telehealth visits were classified as: telephone-not further specified, synchronous video, and asynchronous text message (see Supplement 2, Supplemental Digital Content, https://links.lww.com/MD/P435, Clinic stop code names). Clinic stop code names that were ambiguous were assessed in the electronic health record by evaluation of 5 visits to determine if the visits were conducted consistent with telehealth, and only clinic stop codes without in-person assessment were included. Likewise, primary care visits were identified by review of primary and/or secondary clinic stop codes with chart validation for ambiguous clinic stops in primary care, urgent care, medicine, and women’s clinic that did not include telehealth terms. Stop codes for the Emergency Department were excluded as they may represent higher acuity of illness.^[26,27,29]^

The clinical outcome was the combination of a new outpatient visit or hospitalization that occurred between 3 to 30 days after antibiotic prescription release (i.e., dispensed) date in which the subsequent visit had ICD-10 documentation consistent with a UTI or, if hospitalized ICD-10 documentation of a UTI or complicating genitourinary condition with a suspected urinary source (e.g. sepsis ICD-10 code PLUS UTI code) in which any systemically administered antibiotic was prescribed (see Supplement 3, Supplemental Digital Content, https://links.lww.com/MD/P435, Codes and antibiotics used to define failure).^[26,30]^ To compare the clinical outcome between treatment in telehealth and primary care settings, the first qualifying patient-visit (i.e., each patient was evaluated once)was selected and the propensity to have been seen in telehealth was estimated with a logistic regression model (outcome of telehealth or primary care) developed from factors deemed potentially associated with treatment setting.^[31]^ These included race, ethnicity, age, gender, indicators of chronic comorbidity (Charlson index), inpatient hospitalization within the prior 90 days, time represented as a binary variable indicating pre/post COVID-19 onset (e.g., mid-March, 2020), geographic region (Veterans Integrated Service Network [VISN]), and VA facility complexity as an indicator of size and structure of the treating facility.^[32,33]^ Vital signs were not included due to the limited data availability (~15%) in the telehealth setting. Assuming some degree of similarity among patients within a facility, a generalized estimating equation (GEE) framework using an exchangeable correlation structure was employed.^[34,35]^ Overlap weights were calculated from model outputs and balance was checked (standardized mean difference and variance ratio) across variables used in the propensity models.^[36,37]^ These weights were applied to each patient visit with the goal of balancing the 2 patient groups (e.g., telehealth, primary care) with respect to the potential confounders (e.g., why patients were seen in telehealth or primary care).

The overlap weights were applied in the comparison of clinical failure. This analysis used a Poisson regression, with a log link to facilitate calculation of relative risk.^[38,39]^ Telehealth was modeled relative to Primary Care. Variables in addition to those used in the propensity model included: ICD-10 coded UTI diagnosis [Cystitis, Pyelonephritis, and other unspecified UTI (UTI-not otherwise specified (NOS))], antibiotic prescribed, antibiotic-resistant risk factors identified within the prior 90 days (prescription of study antibiotic/antibiotic class of interest, recovery of antibiotic resistant organism from culture, hospitalization or nursing home residence), and duration of treatment. To facilitate interpretation, age was categorized as < 50 years, 60 to 64 years, and > 65 years, and treatment duration was categorized as ≤7 days, 8 to 10 days, 11 to 14 days, and 15 to 30 days. To further understand whether treatment failure would differ across treatment settings, interactions were fit between setting and time period, age, sex, and antibiotic resistance risk factor indicators.

To evaluate whether collection of UC obtained −1 to 3 days after the index visit impacted clinical outcome in patients with antibiotic resistance risk factors a similar approach was taken. The cohort was divided into 2 distinct sub-cohorts based on treatment setting. Next, overlap weights for the propensity to obtain a UC was modeled for each sub-cohort from the following predictors: race, ethnicity, age, gender, Charlson index, inpatient hospitalization within the prior 90 days, time pre/post COVID onset, ICD-10 coded UTI diagnosis, antibiotic-resistant risk factors, VISN, and VA facility complexity. As before, this propensity model was developed based on the first visit per patient with UC collection as the outcome using logistic regression fit with GEE. to account for correlation among patients in the same VA facility. The same predictors used in development of propensity models were used to calculate the relative risk of clinical failure in each sub-cohort. UC collection was modeled relative to no collection. Interactions were fit between culture collection and age, sex, and antibiotic resistance factors. For each sub-cohort, treatment failure was modeled in a Poisson regression using overlap weights and GEE for patients clustered within facilities.

For all sets of models, E-values were calculated to address the magnitude of unidentified confounders which could potentially impact conclusions regarding treatment setting and collection of UC.^[39]^

All models were built using R version 4.3.1 with RStudio, and using the libraries tidyverse, geepack, cobalt, gt summary and gt, and E-values.^[40–46]^

A post hoc analysis of culture-positive patients including treatment discordance (i.e., culture results indicating non-susceptibility to the antibiotic/antibiotic class prescribed) was conducted. The results were stratified by interactions of interest: patient age ≥65 versus < 65 years of age and by the prior exposure to an antibiotic of interest within the prior 90 days (i.e., antibiotic resistance risk factor). Two-group *t* tests and Chi square tests of independence were used to compare groups.

## 3. Results

**Figure 1.**
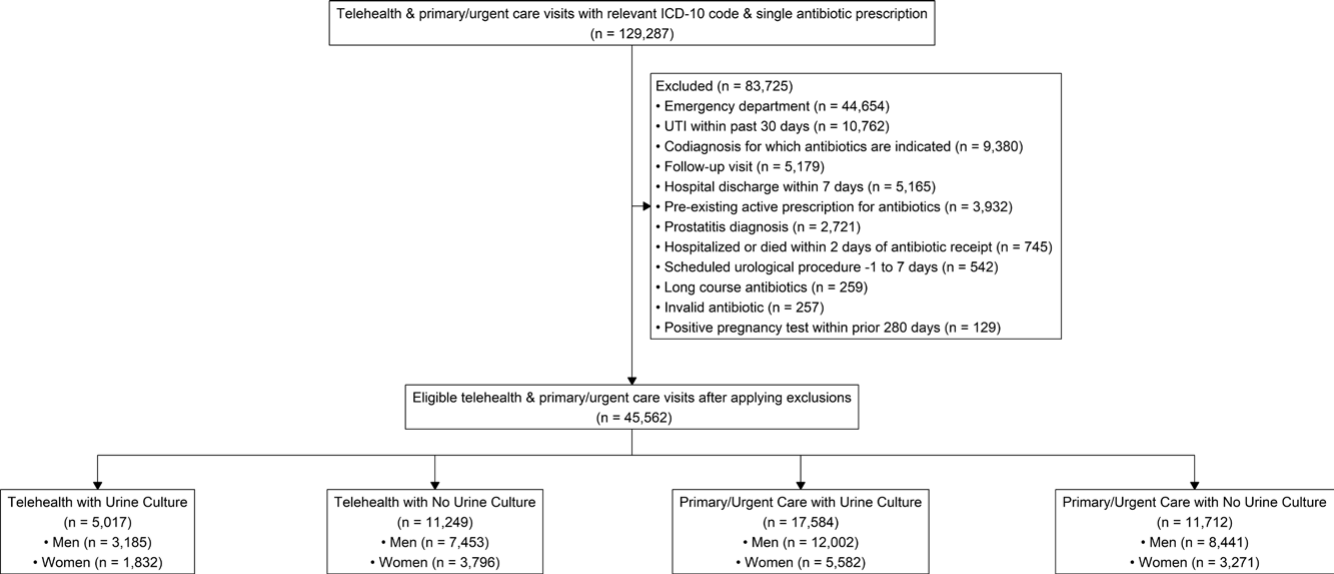
Study flow diagram for Veterans treated for urinary tract infection in telehealth and primary care settings with and without a urine culture collected.

A total of 129,287 outpatient UTI patient-visits within 130 VA medical centers were identified and 45,562 patient-visits were analyzed after applying exclusions (Fig. 1). Patient-visits were administratively coded as: UTI-NOS 42,236 (92.7%), cystitis 3053 (6.7%), and pyelonephritis 273 (0.6%). There were 16,266 (35.7%) patients treated in telehealth including 13,773 (84.7%) telephone-not further specified, 2028 (12.5 %) video visits, and 465 (2.9%) with secure messaging. There were 29,296 (64.3%) patients treated in primary care. The most common antibiotic treatment was nitrofurantoin (31%) and >98% of patients were treated for < 15 days. Patients seen in telehealth were generally younger, female, Hispanic, and had less comorbidity (Table 1). The unadjusted 30-day failure rate was 8.3% for telehealth and 9.4% for primary care.

**Table 1:** Characteristics of outpatients diagnosed with urinary tract infection treated in telehealth and primary care settings.*

Characteristic	Total N = 45,562	Telehealth, N = 16,266	Primary care N = 29,296
Age (y), mean (SD)	65.1 (16.0)	64.5 (16.1)	65.5 (15.9)
Age ≥65 years (%)	58.3	56.2	59.4
Male (%)	68.2	65.4	69.8
Race (%)			
Caucasian	68.9	67.3	69.7
African American	20.2	21.2	19.6
Other	11.0	11.5	10.7
Hispanic/Latino (%)	7.5	8.6	6.9
Charlson Comorbidity, median (IQR)	1.0 (0.0–2.0)	1.0 (0.0–2.0)	1.0 (0.0–2.0)
Urinalysis collected (%)†	58.6	34.8	71.8
Urine culture collected (%)	49.6	30.8	60.0
Antibiotic resistance risk factors (%)‡			
Inpatient stay in ≤90 d	4.3	4.5	4.2
UTI antibiotic exposure in ≤90 d	11.1	11.9	10.6
Antibiotic resistant organism in ≤90 d	7.2	10.8	5.2
Treatment (%)			
Fluoroquinolones	20.7	19.3	21.5
TMP/SMX	26.0	25.4	26.3
Nitrofurantoin	31.0	32.3	30.3
β-lactams	22.3	23.0	21.8
Treatment duration (%)			
** **≤7 d	69.0	71.5	67.6
** **8–10 d	25.4	22.9	26.8
** **11–14 d	4.0	4.0	4.0
** **15–30 d	1.5	1.6	1.5
30-day UTI clinical failure rate (%)			
Aggregate	9.4	8.3	10.0
Outpatient revisit	8.3	7.1	9.0
Hospitalization	1.0	1.1	1.0

D = day, IQR = interquartile range, SD = standard deviation, TMP/SMX = trimethoprim/sulfamethoxazole, β-lactams included cephalexin, cefuroxime, cefpodoxime, cefdinir, amoxicillin/clavulanate, amoxicillin, UTI = urinary tract infection, Y = year.

* Demographic characteristics for cohort after exclusions applied (Fig. 1).

† Includes 102 patients with specimens definitively collected via catheterization.

‡ Adapted from references.^[2,5,6,26,47]^

**Figure 2.**
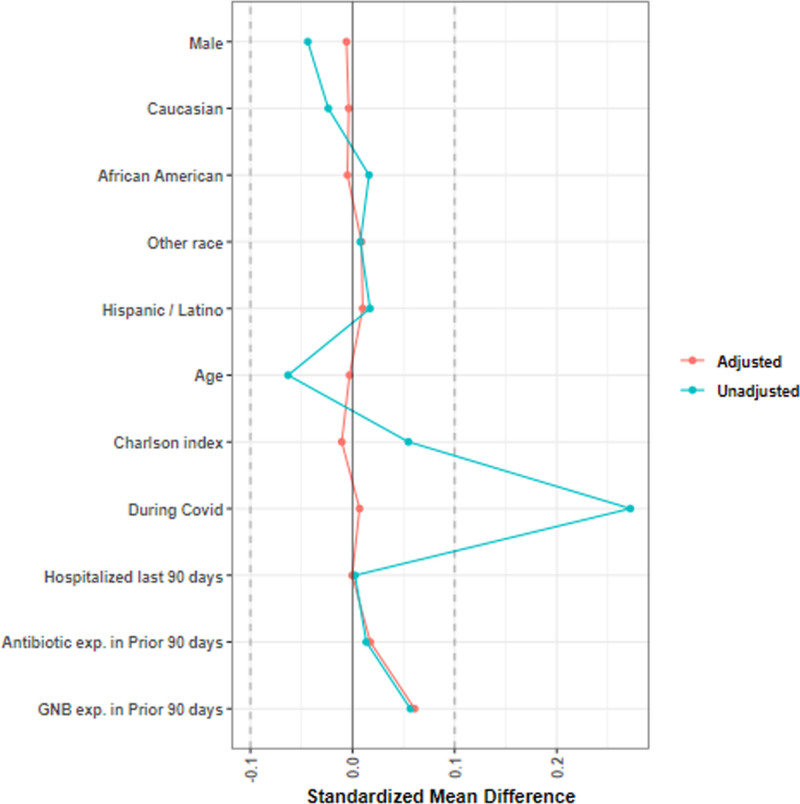
Overlap weighting balance plot for Veterans treated for urinary tract infection in telehealth and primary care settings.

After development of the overlap weighting models, potential confounders in setting treatment groups were well balanced (Fig. 2).

The adjusted relative risk (±95% CI) of 30-day clinical failure for telehealth relative to primary care was [0.87 (0.70, 1.08)] (Table 2). Notable covariates associated with increased risk for failure included higher Charlson score, hospitalization or antibiotic exposure within ≤90 days, treatment with antibiotics other than fluoroquinolones, and shorter duration of treatment. An interaction between setting and age ≥65 years [1.45 (1.12, 1.87)] relative to < 65 years identified higher risk for a worse outcome among elderly patients treated in telehealth. The E-value was 1.6 with a lower bound of 1.0 for pre COVID-19 and 2.3 with a lower bound of 1.7 post COVID-19.

**Table 2:** Relative risk (± 95% CI) of clinical failure for outpatient veterans treated for urinary tract infection in telehealth and primary care settings.

Term	Relative risk (± 95% CI)
(Intercept)	0.08 (0.06, 0.10)
Telehealth (relative to primary care)	**0.87 (0.70, 1.08**)
Men (relative to women)	**1.31 (1.16, 1.47**)
Race (relative to Caucasian)	
African American	0.93 (0.85, 1.01)
Other	0.96 (0.86, 1.07)
Hispanic/Latino	**0.86 (0.77, 0.96**)
Age (relative to < 50 y)	
50–64 y	1.05 (0.92, 1.19)
≥65 y	0.92 (0.72, 1.07)
Charlson Index (Scaled)	**1.16 (1.13, 1.19**)
Time (per/post COVID initiation)	1.01 (0.94, 1.10)
Antibiotic resistance risk factors	
Antibiotic exposure within ≤90 d	**1.70 (1.52, 1.90**)
Resistant organism within ≤90 d	0.94 (0.84, 1.10)
Inpatient stay within ≤90 d	**1.32 (1.16, 1.52**)
Treatment (relative to Fluoroquinolone)	
β-Lactam	**1.50 (1.35, 1.68**)
Nitrofurantoin	**1.39 (1.26, 1.54**)
TMP/SMX	**1.21 (1.08, 1.36**)
UTI ICD-10 Code (relative to UTI-NOS)	
Cystitis	1.02 (0.91, 1.15)
Pyelonephritis	1.21 (0.80, 1.82)
Treatment duration (relative to ≤ 7 d)	
8–10 d	**0.92 (0.86, 0.98**)
11–14 d	0.93 (0.78, 1.03)
>14 d	**0.70 (0.52, 0.95**)
Interactions with telehealth	
Time period	**0.79 (0.69, 0.90**)
Age category (relative to <50 y)	
50–64 y	1.26 (0.96, 1.51)
≥65 y	**1.45 (1.12, 1.87**)
Men (relative to women)	**0.83 (0.70, 0.99**)
Antibiotic resistance risk factors	
Antibiotic exposure within ≤90 d	0.99 (0.83, 1.19)
Resistant organism within ≤90 d	0.91 (0.74, 1.14)
Inpatient stay within ≤90 d	0.97 (0.79, 1.19)

Model also adjusted for VA facility complexity and Veterans Integrated Service Network (VISN) region (data not shown). RR = relative risk, 95% CI = ±95% confidence intervals. Significance based on generalized estimating equations (GEE), Poisson distribution with log link (to return relative risk), Clustering on facility. Interaction RR is ratio or relative risks. All RR represent 1-unit change. Sample sizes utilized in calculating interactions for treatment setting included: age: telehealth ≤50 (n = 3164), 50–64 (n = 3962), ≥65 (n = 9143); primary care ≤50 (n = 5043), 50–64 (n = 6856), ≥65 (n = 17,397), sex: telehealth – female (n = 5628), male (n = 10,638); primary care – female (n = 8853), male (n = 2),443, antibiotic exposure ≤90 days: telehealth – yes (n = 1945), No (n = 14,321); primary care – yes (n = 3108), no (n = 26,188), antibiotic resistant organism ≤90 days: telehealth – yes (n = 1762), no (n = 14,504); primary care – yes (n = 1514), no (n = 27,782), inpatient stay ≤90 days: telehealth – yes (n = 726), no (n = 15,540); primary care – yes (n = 1238), no (n = 28,058). Values highlighted in bold indicate statistical significance.

D = day, IQR = interquartile range, SD = standard deviation, TMP/SMX = trimethoprim/sulfamethoxazole, UTI-NOS = UTI not otherwise specified, Y = year, β-lactams included cephalexin, cefuroxime, cefpodoxime, cefdinir, amoxicillin/clavulanate, amoxicillin.

**Figure 3.**
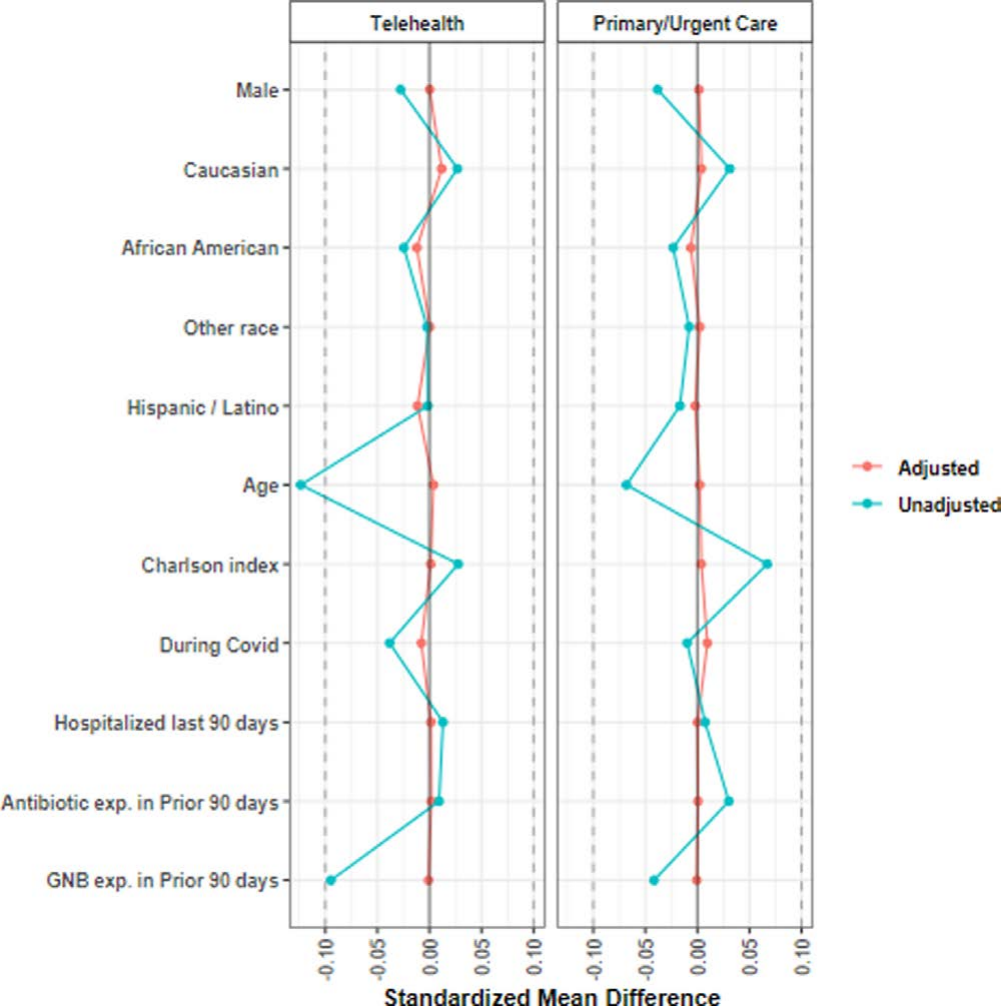
Overlap weighting balance plot for Veterans treated for urinary tract infection in telehealth and primary care settings with and without a urine culture collected.

UC were collected in 30.8% of telehealth and 60.0% of primary care patient-visits (Table 1). After development of the overlap weighting models for separate treatment settings, potential confounders of culture collection available were well balanced (Fig. 3). UC collection was associated with an increased risk of failure treated in telehealth [2.06 (1.56, 2.72)] but not primary care [1.17 (0.93, 1.47)] settings (Table 3). Other significant predictors of clinical failure in models that included UC collection were increased age in telehealth setting, higher Charlson score, hospitalization or antibiotic exposure within ≤90 days, treatment with antibiotics other than fluoroquinolones, and shorter duration of treatment. An interaction was observed in which UC collection was associated with a reduced risk of clinical failure in patients with a recent antibiotic exposure (≤90 days) in both telehealth [0.70 (0.53, 0.93)] and primary care [0.77 (0.60, 0.99)] settings. The E-value from the telehealth sub-cohort was 3.5 (lower bound 2.5) and from the primary care sub-cohort was 1.6 (lower bound 1.0).

**Table 3:** Relative risk (+ 95% CI) of urinary tract infection clinical failure for patients with and without a urine culture obtained.

Term	Telehealth	Primary care
(Intercept)	0.04 (0.02, 0.06)	0.07 (0.05, 0.10)
Urine culture collected	**2.06 (1.56, 2.72**)	1.17 (0.93, 1.47)
Men (relative to women)	1.08 (0.89, 1.31)	**1.25 (1.06, 1.48**)
Race (relative to Caucasian)		
African American	0.91 (0.76, 1.09)	0.97 (0.89, 1.06)
Other	1.04 (0.86, 1.25)	1.00 (0.89, 1.12)
Hispanic/Latino	0.86 (0.73, 1.01)	0.87 (0.73, 1.04)
Age (relative to <50 y)		
50–64 y	**1.38 (1.04, 1.81**)	0.83 (0.68, 1.08)
≥65 y	**1.37 (1.04, 1.82**)	**0.66 (0.52, 0.84**)
Charlson Index (scaled)	**1.17 (1.13, 1.19**)	**1.15 (1.12, 1.19**)
Time (pre/post COVID onset)	**0.85 (0.74, 0.97**)	1.02 (0.95, 1.09)
Antibiotic resistance risk factors		
Antibiotic exposure within ≤90 d	**1.90 (1.59, 2.28**)	**2.00 (1.61, 2.47**)
Resistant organism within ≤90 d	1.06 (0.90, 1.25)	1.05 (0.84, 1.31)
Inpatient stay within ≤90 d	**1.31 (1.01, 1.70**)	**1.58 (1.25, 2.01**)
Treatment (relative to Fluoroquinolone)		
Beta-Lactam	**1.64 (1.35, 2.01**)	**1.38 (1.22, 1.55**)
Nitrofurantoin	**1.54 (1.29, 1.83**)	**1.27 (1.13, 1.42**)
TMP/SMX	**1.37 (1.13, 1.66**)	**1.13 (1.01, 1.27**)
UTI ICD-10 Code (relative to UTI-NOS)		
Cystitis	0.97 (0.77, 1.23)	1.09 (0.96, 1.25)
Pyelonephritis	0.37 (0.09, 1.53)	1.09 (0.76, 1.58)
Treatment duration (relative to ≤7 d)		
8–10 d	0.89 (0.79, 1.01)	**0.93 (0.86, 1.00**)
11–14 d	1.05 (0.79, 1.31)	0.95 (0.80, 1.13)
>14 d	0.88 (0.56, 1.37)	**0.64 (0.45, 0.93**)
Interactions with urine culture collection		
Age category (relative to <50 y)		
50–64 y	0.84 (0.56, 1.26)	1.35 (0.98, 1.86)
≥65 y	0.99 (0.66, 1.48)	**1.53 (1.17, 2.01**)
Men (relative to women)	1.06 (0.81, 1.39)	1.05 (0.86, 1.28)
Antibiotic resistance risk factors		
Antibiotic exposure within ≤90 d	**0.70 (0.53, 0.93**)	**0.77 (0.60, 0.99**)
Resistant organism within ≤90 d	0.75 (0.54, 1.05)	1.09 (0.83, 1.44)
Inpatient stay within ≤90 d	1.04 (0.70, 1.54)	0.82 (0.61, 1.09)

Models also adjusted for VA facility complexity and Veterans Integrated Service Network (VISN) region (data not shown). Significance Based on GEE, Poisson distribution with log link (to return relative risk), Clustering on facility. Interaction RR is ratio or relative risks. All RR represent 1-unit change. Sample sizes utilized in calculating interactions for urine culture collection included: telehealth: age: culture ≤50 (n = 1066), 50–64 (n = 1315), ≥65 (n = 2636); no culture ≤50 (n = 2095), 50–64 (n = 2647), ≥65 (n = 6507) sex: culture – female (n = 1832), male (n = 3185); no culture – female (n = 3796), male (n = 7453), antibiotic exposure ≤90 days: culture – yes exposure (n = 632), no exposure (n = 4385); no culture – yes exposure (n = 1313), no exposure (n = 9936), ARO ≤90 days: culture – yes prior ARO (n = 215), no prior ARO (n = 4802); no culture – yes prior ARO (n = 1547), no prior ARO (n = 9702), inpatient stay ≤90 days: Culture – yes inpatient stay (n = 269), no inpatient stay (n = 4748); no culture – yes inpatient stay (n = 457), no inpatient stay (n = 10,792). Primary care: age: culture ≤50 (n = 3185), 50–64 (n = 4130), ≥65 (n = 10,269); no culture ≤50 (n = 1858), 50–64 (n = 2726), ≥65 (n = 7128) sex: culture – female (n = 5582), male (n = 12,002); no culture – female (n = 3271), male (n = 8441), antibiotic exposure ≤90 days: culture – yes exposure (n = 2078), no exposure (n = 15,506); no culture – yes exposure (n = 1030), no exposure (n = 10,682), ARO ≤90 days: culture – yes prior ARO (n = 616), no prior ARO (n = 16,968); no culture – yes prior ARO. Values highlighted in bold indicate statistical significance.

ARO = antibiotic resistant organism, 95% CI = ±95% confidence intervals, D = day, IQR = interquartile range, RR = relative risk, SD = standard deviation, TMP/SMX = trimethoprim/sulfamethoxazole, UTI-NOS = UTI not otherwise specified, Y = year, β-lactams included cephalexin, cefuroxime, cefpodoxime, cefdinir, amoxicillin/clavulanate, amoxicillin.

In aggregate, 12,156 (53.8%) of UC collected had culture and susceptibility results reported (Table 4). *Escherichia coli* was reported in 54.4% of positive cultures followed by other Gram-negative bacilli and Gram-positive cocci. Culture-treatment discordance occurred in 1076 (8.9%) of patient-visits primarily due to non-*E coli* Gram-negative bacilli treatment-susceptibility mismatches. Patients ≥65 years of age were more likely to be culture-positive than younger patients in both settings. Culture-positive patients ≥65 were more likely to exhibit discordance than younger patients in telehealth but not in primary care. Recent prior exposure to an antibiotic of interest was present in 1584 (13.0%) of culture-positive patients. Culture-treatment discordance occurred in 10.5% with recent prior antibiotic exposure versus 8.6% in patients without recent prior exposure. Based on this ad hoc analysis, the number needed to culture to identify treatment discordance for patients ≥65 years of age was 11 and for recent prior antibiotic exposure was 10.

**Table 4:** Culture-positive results and treatment discordance stratified by urine culture interaction.

Description	All settings (*N*, %) (22,599, 49.6)	Telehealth (*N*, %) (5010, 54.1)	Primary care (*N*, %) (9446, 53.7)
Positive urine cultures	12,156 (53.8)	2710	9446
* E coli*	6607 (54.4)	1516 (55.9)	5091 (53.9)
Other GNB	3634 (29.9)	803 (29.6)	2831 (30.0)
GPC*	1915 (15.8)	391 (14.4)	1524 (16.1)
Treatment-culture discordance	1076 (8.6)	235 (8.7)	841 (8.9)
* E coli*	458 (6.9)	100 (6.6)	358 (7.0)
Other GNB	537 (14.8)	122 (15.2)	415 (14.7)
GPC	81 (4.2)	13 (3.3)	68 (4.5)
Age			
<65 y with positive culture†	4618 (38.0)	1126 (43.0)	3492 (37.8)
≥65 y with positive culture	7234 (59.6)	1492 (57.0)	5742 (62.2)
Age and discordance			
Discordance <65 y	414 (9.0)	84 (7.5)	330 (9.5)
Discordance ≥65 y	656 (9.1)	149 (10.0)	507 (8.8)
Recent prior antibiotic exposure			
No antibiotic exposure <90 d	10,572 (87.0)	2344 (86.5)	8228 (87.1)
Antibiotic exposure <90 d	1584 (13.0)	366 (13.5)	1218 (12.9)
Prior antibiotics and discordance			
Discordance W/O prior exposure	910 (8.6)	194 (8.3)	125 (10.3)
Discordance W/ prior exposure	166 (10.5)	41 (11.2)	716 (8.7)

Positive urine culture = reported organism plus sensitivity to at least 1 antibiotic/ antibiotic class reported. Definitions modified to include antibiotic exposure to any of the antibiotics of interest (nitrofurantoin, trimethoprim/sulfamethoxazole, ciprofloxacin, levofloxacin, cephalexin, cefuroxime, cefpodoxime, cefdinir, amoxicillin/clavulanate, amoxicillin) and organisms with non-susceptibility results reported to fluoroquinolones, 3rd or 4th generation cephalosporins, nitrofurantoin, or trimethoprim/sulfamethoxazole. In select cases CLSI expert rules were applied (e.g. MRSA considered resistant to all B-lactams, *E coli* resistant to ciprofloxacin also considered resistant to levofloxacin, etc) Other GNB = gram-negative bacilli include: *Klebsiella spp*. (15.0%), *Proteus spp*. (6.4%), *Citrobacter spp*. (2.9%), *Enterobacter spp*. (2.2%), *Pseudomonas aeruginosa* (2.2%) and other (2.9%), GPC = gram-positive cocci include: *Enterococcus spp* (8.9%), Coagulase-negative Staphylococcus (4.2%), *Staphylococcus aureus* (2.6%) and other (1.1%), Y = years of age, W = with, W/O = without, prior antibiotic exposure defined by prescription fill for any antibiotic of interest.

* Patients using primary care have significantly more positive GPC cultures than telehealth patients (T_1_ = 4.62, *P* = .032).

† There are significantly more patients under 65 years with a positive culture in TH than expected by chance alone (*X*^2^_1_ = 23.1, *P* < .0001).

## 4. Discussion

This study identified several important findings regarding treatment of UTI in telehealth settings. First, outcome of treatment for outpatient UTI in this predominantly elderly, mixed sex cohort with moderate comorbidity was not worse in telehealth compared to in-person primary care. However, patients ≥65 years of age exhibited a higher failure rate relative to younger adults treated in telehealth settings. Male sex and chronic comorbidity were associated with increased risk of failure. Hispanic ethnicity was associated with reduced risk of failure; however, there was a higher frequency of Hispanic young females in the cohort than non-Hispanic males which likely explains these findings. As anticipated, the presence of antibiotic resistance risk factors was associated with a higher risk for clinical failure.^[2–6,26,27,47]^ In a prior evaluation of in-person emergency department and primary care visits we identified that antibiotic treatment selection and duration, as well as antibiotic resistance risk factors were important variables associated with clinical outcome in the Veteran population and findings from the current study generally parallel those observations.^[26,27]^ UC collection was associated with an increased risk for failure in the telehealth setting. UC were collected twice as frequently in the primary care setting and it is likely that cultured telehealth patients differed from patients without cultures obtained. Inspection of interactions between UC collection and antibiotic resistance risk factors identified a protective effect for obtaining a UC in patients with recent prior antibiotic exposure, irrespective of treatment setting. The study definitions for antibiotic resistance risk factors differ slightly from some defined in guideline recommendations but are similar to others.^[2–6]^ In actuality, recent prior antibiotic exposures and recovery of antibiotic resistant organisms to UTI antibiotics of interest are highly correlated, although the latter may be more generalizable in the outpatient setting.^[47]^ In general, the findings support the collection of UC for patients with recent prior antibiotic exposure in outpatients treated for UTI.

Considerations for application of findings include understanding the reasons underlying the increased UTI failure rate in elderly patients and the decreased failure rate associated with UC collection in patients with recent prior antibiotic exposure. Potential reasons for a higher return visit rate for elderly patients in telehealth include miscommunication, misdiagnosis, or suboptimal treatment selection. Prior analyses have identified elderly patients as being at increased risk for complications of UTI care, including suboptimal selection of therapy.^[48,49]^ In the current analysis, patients ≥65 did not have a higher discordance rate than younger patients making this explanation by itself less likely. However, culture-positive patients made up only 1/4th of the cohort. Given the high proportion of male patients with comorbidity, misdiagnosis is a possible explanation, particularly in the absence of vital signs. Digital literacy in elderly patients may also contribute to miscommunication view telehealth platforms and many Veterans receiving telehealth primary care have expressed an interest in technology training to facilitate telehealth visits.^[50]^ Health systems should consider structuring platforms to collect additional information on urologic comorbidities, prior antibiotic exposures, and digital literacy as part of their screening process. Then considering recommendation of in-person care for patients > 65, particularly if they have potential for complicating conditions, antibiotic resistant infection, or express concerns with telehealth care. Minimally, telehealth programs should design protocols for appropriate collection of UC in symptomatic patients with recent prior antibiotic exposures. This is particularly important in the era of diagnostic stewardship in which many health systems are trying to reduce the collection of UA and reflex UC to decrease the treatment of asymptomatic bacteriuria.^[8,9]^

Study strengths include the large nationwide cohort of detailed integrated outpatient and inpatient medical records for a mixed sex population of patients with UTIs. Index visits were limited to those conducted through telehealth or in-person non-Emergency Department visits in which recent prior visits for UTI, competing infectious diagnoses where antibiotic treatment may have been indicated, and other complicating genitourinary conditions were excluded.^[27,29]^ A real world definition of clinical failure which required documentation of a new encounter for UTI between 3 to 30 days post initial treatment plus a new antibiotic prescription was utilized. Irrespective of whether the revisit cause was a relapse, recurrent infection, or for a different reason (e.g., misdiagnosis, adverse event) this definition reflected the delivery of further care. A final strength included adjustment using overlap weighting which creates exact balance on the mean of measured covariates.^[36,37]^

A key study limitation is the lack of illness acuity which might influence the choice to seek telehealth or in-person care; the absence of which may limit interpretability and generalizability of the findings. Most telehealth visits lacked vital sign documentation in the CDW, leaving these illness indicators unexamined. Also, we did not directly collect patient-level data on rurality or residence distance from in-person care locations which may have impacted UC collection; although these data were indirectly factored into VA facility complexity scores. These data could, if incorporated into the outcome models, potentially alter the conclusions. The lower bound of the E-value (1.6) for treatment failure during COVID-19 is within the range of relative risks observed for treatment failure which indicates that an unmeasured confounder could alter conclusions with respect to the telehealth setting. For sub-cohort models, the lower bound of the E-value (2.5) exceeded the effect of all measured covariates in the telehealth model, suggesting that it is unlikely that an unmeasured covariate could result in a different conclusion regarding culture collection in this setting.

The study has additional limitations consistent with retrospective database studies. Patients could have received diagnostic testing or treatment in non-VA settings which would not be completely captured. Second, UTI index visits were identified utilizing administrative data and it is possible that some patients did not have a UTI. The algorithm used to identify cases has demonstrated good sensitivity to detect a clinician’s *intent* to treat a UTI; however, the specificity to detect complicated infection based on administrative coding (e.g., cystitis, pyelonephritis) within the VA and elsewhere is poor as most diagnoses are coded as UTI-NOS.^[24,26,27,29]^ Further, while we excluded patients with concurrent urological procedures we were only able to identify a small number of patients with catheters, which could indicate a complicated infection. The availability of catheter data was limited within the CDW and natural language processing would be required to obtain catheter utilization data from clinician notes which was beyond the scope of this analysis. The study time-frame included both pre/post COVID-19 periods. COVID-19 resulted in an increase in telehealth and Emergency Department visits and a decrease in primary care visits which may have impacted the case-mix of patients receiving care in both settings. Finally, reverse causation in which sicker patients were more likely to have a UC collected but also more likely to experience increased clinical failure cannot be excluded. Causality cannot be inferred due to the retrospective study design.

A number of studies have compared the treatment of UTI between telehealth and in person settings; however almost all have limited their study populations to uncomplicated UTI.^[12–17]^ Studies of predominantly uncomplicated infection have identified slight improvements in outcome in telehealth or equivalency with fewer diagnostic tests ordered and higher use of guideline recommended treatments. Limited studies have included or evaluated complicated UTI.^[18–20,49]^ Daumier et al retrospectively evaluated outcomes associated with a national UTI telemedicine program that included over 51,000 patients including over 17,000 with potentially complicated UTI.^[19]^ A subset of patients was evaluated for symptoms at 7 days and revisit at 30 days. Non-significant differences in clinical cure were observed with 90.8% of uncomplicated UTI vs 87.9% for complicated UTI. DeWitt-Foy et al evaluated the impact of telehealth on urine testing, antibiotic prescription patterns, and UTI outcomes in 6744 patients in a large academic healthcare system.^[18]^ They identified that UC were obtained in 19% of telehealth patients and 70% of in-person visits and that UC collection was not associated with complicated UTI. Telehealth was associated with an increase in 14-day UTI revisits and a slight increase for new antibiotic prescriptions, although no change in hospitalization. Complicated UTI and age > 50 relative to age 19 to 29 was associated with an increase in return visits; however, the analysis did not report if this finding was specific to telehealth. Another large analysis of health plans with direct to consumer health plan coverage compared to those without found no difference in the need for follow-up between telehealth versus primary care or for patients > 50 years of age compared to those younger than 50.^[20]^ Rather than focus on classifying UTI as complicated or uncomplicated we evaluated utility of UC collection based on the presence or absence of antibiotic resistance risk factors.^[2–6,25,26,47]^ In a multi-centered utilization review of 3250 outpatients with positive UCs, we previously identified that discordant therapy was prescribed in approximately 20% of patients, and that antibiotic resistance risk factors as defined were present in 19% of patients.^[26]^ The current study adds further support for collecting UC in all patients with these risk factors in the past 90 days, particularly prior antibiotic exposure.

In conclusion, this study found that UTI treatment in telehealth settings was effective in a large mixed sex cohort of Veterans; however, elderly patients had an increased risk for clinical failure relative to younger patients. While UC were obtained infrequently in telehealth settings, collection from patients with recent prior antibiotic exposure regardless of treatment setting was associated with a reduced risk for clinical failure. Health systems that utilize telehealth for treatment of UTI in elderly patients should consider development of tailored protocols for this population, particularly those with risk factors for complications or limited digital literacy. Further, telehealth treatment pathways should consider recent prior antibiotic exposure when assessing the need to obtain UC.

## Acknowledgments

The findings and conclusions in this report are those of the authors and do not necessarily represent the official position of the Department of Veterans Affairs.

## Author contributions

**Conceptualization:** Karl J. Madaras-Kelly, Jeremy K. Boyd.

**Data curation:** Jeremy K. Boyd.

**Formal analysis:** Laura Bond.

**Funding acquisition:** Karl J. Madaras-Kelly.

**Investigation:** Karl J. Madaras-Kelly, Laura Bond.

**Methodology:** Karl J. Madaras-Kelly, Jeremy K. Boyd, Laura Bond.

**Project administration:** Karl J. Madaras-Kelly.

**Resources:** Karl J. Madaras-Kelly.

**Supervision:** Karl J. Madaras-Kelly.

**Validation:** Karl J. Madaras-Kelly, Jeremy K. Boyd.

**Visualization:** Jeremy K. Boyd, Laura Bond.

**Writing – original draft:** Karl J. Madaras-Kelly.

**Writing – review & editing:** Karl J. Madaras-Kelly, Jeremy K. Boyd, Laura Bond.

## Supplementary Material



## References

[1] Anon. Reduce the rate of hospital admissions for urinary tract infections among older adults-OA 07. Healthy People 2030. https://health.gov/healthypeople/objectives-and-data/browse-objectives/infectious-disease/reduce-rate-hospital-admissions-urinary-tract-infections-among-older-adults-oa-07. Accessed February 7, 2025.

[2] Gupta K Hooton TM Naber KG. International clinical practice guidelines for the treatment of acute uncomplicated cystitis and pyelonephritis in women: a 2010 update by the Infectious Diseases Society of America and the European Society for Microbiology and Infectious Diseases. Clin Infect Dis. 2011;52:e103–20. 21292654 10.1093/cid/ciq257

[3] Nelson Z Aslan AT Beahm NP. Guidelines for the prevention, diagnosis, and management of urinary tract infections in pediatrics and adults: a wiki guidelines group consensus statement. JAMA Netw Open. 2024;7:e2444495. 39495518 10.1001/jamanetworkopen.2024.44495

[4] Anon. Public comment IDSA guideline on management and treatment of complicated urinary tract infections. Infectious Diseases Society of America. https://www.idsociety.org/practice-guideline/complicated-uti/. Accessed April 3, 2025.

[5] Gupta K. Acute simple cystitis in male adults. https://www.uptodate.com/contents/acute-simple-cystitis-in-male-adults? Accessed July 10, 2025.

[6] Gupta K. Acute complicated urinary tract infection (including pyelonephritis) in adults. https://www.uptodate.com/contents/acute-complicated-urinary-tract-infection-including-pyelonephritis-in-adults? Accessed July 10, 2025.

[7] Kranz J Schmidt S Lebert C. The 2017 update of the German clinical guideline on epidemiology, diagnostics, therapy, prevention, and management of uncomplicated urinary tract infections in adult patients: part 1. Urol Int. 2018;100:263–70. 29342469 10.1159/000486138

[8] Claeys KC Blanco N Morgan DJ Leekha S Sullivan KV. Advances and challenges in the diagnosis and treatment of urinary tract infections: the need for diagnostic stewardship. Curr Infect Dis Rep. 2019;21:11. 30834993 10.1007/s11908-019-0668-7

[9] Hojat LS Saade EA Hernandez AV Donskey CJ Deshpande A. Can electronic clinical decision support systems improve the diagnosis of urinary tract infections? A systematic review and meta-analysis. Open Forum Infect Dis. 2022;10:ofac691. 36632418 10.1093/ofid/ofac691 PMC9830539

[10] Weiner JP Bandeian S Hatef E Lans D Liu A Lemke KW. In-person and telehealth ambulatory contacts and costs in a large US insured cohort before and during the COVID-19 pandemic. JAMA Netw Open. 2021;4:e212618. 33755167 10.1001/jamanetworkopen.2021.2618 PMC7988360

[11] Barnett ML Huskamp HA Busch AB Uscher-Pines L Chaiyachati KH Mehrotra A. Trends in outpatient telemedicine utilization among rural medicare beneficiaries, 2010 to 2019. JAMA Health Forum. 2021;2:e213282. 35977168 10.1001/jamahealthforum.2021.3282 PMC8727042

[12] Johnson KL Dumkow LE Salvati LA Johnson KM Yee MA Egwuatu NE. Comparison of diagnosis and prescribing practices between virtual visits and office visits for adults diagnosed with uncomplicated urinary tract infections within a primary care network. Infect Control Hosp Epidemiol. 2021;42:586–91. 33118916 10.1017/ice.2020.1255

[13] Murray MA Penza KS Myers JF Furst JW Pecina JL. Comparison of eVisit management of urinary symptoms and urinary tract infections with standard care. Telemed J E Health. 2020;26:639–44. 31313978 10.1089/tmj.2019.0044

[14] Entezarjou A Calling S Bhattacharyya T. Antibiotic prescription rates after eVisits versus office visits in primary care: observational study. JMIR Med Inform. 2021;9:e25473. 33720032 10.2196/25473 PMC8077790

[15] Mehrotra A Paone S Martich GD Albert SM Shevchik GJ. A comparison of care at e-visits and physician office visits for sinusitis and urinary tract infection. JAMA Intern Med. 2013;173:72–4. 23403816 10.1001/2013.jamainternmed.305 PMC3889474

[16] Gordon AS Adamson WC DeVries AR. Virtual visits for acute, nonurgent care: a claims analysis of episode-level utilization. J Med Internet Res. 2017;19:e35. 28213342 10.2196/jmir.6783 PMC5336603

[17] Novara G Checcucci E Crestani A. Telehealth in urology: a systematic review of the literature. How much can telemedicine be useful during and after the COVID-19 pandemic? Eur Urol. 2020;78:786–811. 32616405 10.1016/j.eururo.2020.06.025 PMC7301090

[18] DeWitt-Foy ME Albersheim J Grove S. Impact of virtual care on outpatient urinary tract infection management. Urology. 2023;182:40–7. 37708981 10.1016/j.urology.2023.08.028

[19] Daumeyer NM Kreitzberg D Gavin KM Bauer TA. Real-world evidence: telemedicine for complicated cases of urinary tract infection. PLoS One. 2023;18:e0280386. 36730176 10.1371/journal.pone.0280386 PMC9894494

[20] Yu J Huckfeldt PJ Mink PJ Mehrotra A Abraham JM. Evaluating the association between expanded coverage of direct-to-consumer telemedicine and downstream utilization and quality of care for urinary tract infections and sinusitis. Health Serv Res. 2023;58:976–87. 36622637 10.1111/1475-6773.14129 PMC10480089

[21] Monaghesh E Hajizadeh A. The role of telehealth during COVID-19 outbreak: a systematic review based on current evidence. BMC Public Health. 2020;20:1193. 32738884 10.1186/s12889-020-09301-4 PMC7395209

[22] Anon. National Healthcare Quality and Disparities Report: Chartbook on Healthcare for Veterans. Rockville (MD): Agency for Healthcare Research and Quality (US); 2020 Nov. Overview of VHA patient, veteran, and non-veteran populations and characteristics. https://www.ncbi.nlm.nih.gov/books/NBK578553/. Accessed June 3, 2024. 35263061

[23] Von Elm E Altman DG Egger M Pocock SJ Gøtzsche PC Vandenbroucke JP; STROBE Initiative. The Strengthening the Reporting of Observational Studies in Epidemiology (STROBE) statement: guidelines for reporting observational studies. Lancet. 20079596;370:1453–7. 18064739 10.1016/S0140-6736(07)61602-X

[24] Germanos G Light P Zoorob R. Validating use of electronic health data to identify patients with urinary tract infections in outpatient settings. Antibiotics (Basel). 2020;9:536. 32854205 10.3390/antibiotics9090536 PMC7558992

[25] Fleming-Dutra KE Hersh AL Shapiro DJ. Prevalence of inappropriate antibiotic prescriptions among US ambulatory care visits, 2010-2011. JAMA. 2016;315:1864–73. 27139059 10.1001/jama.2016.4151

[26] Rovelsky SA Vu M Barrett AK. Outpatient treatment and clinical outcomes of bacteriuria in veterans: a retrospective cohort analysis. Antimicrob Steward Healthc Epidemiol. 2022;2:e168. 36483437 10.1017/ash.2022.285 PMC9726514

[27] Madaras-Kelly KJ Boyd J Bond L. Comparative effectiveness of oral antibiotics to treat urinary tract infections in male and female outpatients. Open Forum Infect Dis. 2023;10(Supplement_2):ofad500.2444.

[28] Fihn SD Francis J Clancy C. Insights from advanced analytics at the Veterans Health Administration. Health Aff (Millwood). 2014;33:1203–11. 25006147 10.1377/hlthaff.2014.0054

[29] Samore M Goetz M Nevers M. Tier-based antimicrobial stewardship metrics for genitourinary-related antibiotic use in Veterans’ Affairs outpatient settings. Antimicrob Steward Healthc Epidemiol. 2022;2(Suppl 1):s5–6.

[30] Søgaard KK Thomsen RW Schønheyder HC Søgaard M. Positive predictive values of the International Classification of Diseases, 10th revision diagnoses of Gram-negative septicemia/sepsis and urosepsis for presence of Gram-negative bacteremia. Clin Epidemiol. 2015;7:195–9. 25709502 10.2147/CLEP.S75262 PMC4334314

[31] Austin PC Stuart EA. Moving towards best practice when using inverse probability of treatment weighting (IPTW) using the propensity score to estimate causal treatment effects in observational studies. Stat Med. 2015;34:3661–79. 26238958 10.1002/sim.6607 PMC4626409

[32] Glasheen WP Cordier T Gumpina R Haugh G Davis J Renda A. Charlson comorbidity index: *ICD-9* update and *ICD-10* translation. Am Health Drug Benefits. 2019;12:188–97. 31428236 PMC6684052

[33] Anon. National Academies of Sciences, Engineering, and Medicine. Facilities staffing requirements for the Veterans Health Administration—resource planning and methodology for the future: interim report. https://nap.nationalacademies.org/read/25455/chapter/2. Accessed June 3, 2024. 32293829

[34] Hardin J Hilbe JM. Generalized estimating equations by Hardin JW, HILBE, J. M. Biometrics. 2013;69:799.

[35] Liang KY Zeger SL. Longitudinal data analysis using generalized linear models. Biometrika. 1986;73:13–22.

[36] Li F Thomas LE Li F. Addressing extreme propensity scores via the overlap weights. Am J Epidemiol. 2019;188:250–7. 30189042 10.1093/aje/kwy201

[37] Thomas LE Li F Pencina MJ. Overlap weighting: a propensity score method that mimics attributes of a randomized clinical trial. JAMA. 2020;323:2417–8. 32369102 10.1001/jama.2020.7819

[38] Austin PC. An introduction to propensity score methods for reducing the effects of confounding in observational studies. Multivariate Behav Res. 2011;46:399–424. 21818162 10.1080/00273171.2011.568786 PMC3144483

[39] Chen W Qian L Shi J Franklin M. Comparing performance between log-binomial and robust Poisson regression models for estimating risk ratios under model misspecification. BMC Med Res Methodol. 2018;18:63. 29929477 10.1186/s12874-018-0519-5 PMC6013902

[40] VanderWeele TJ Ding P. Sensitivity analysis in observational research: introducing the E-value. Ann Intern Med. 2017;167:268–74. 28693043 10.7326/M16-2607

[41] R Core Team. R: a language and environment for statistical computing. Vienna, Austria; 2023. https://www.R-project.org/. Accessed June 7, 2024.

[42] Posit team. RStudio: integrated development environment for R. Boston, MA; 2023. http://www.posit.co/. Accessed June 7, 2024.

[43] Wickham H Averick M Bryan J. Welcome to the tidyverse. J Open Source Softw. 2019;4:1686.

[44] Halekoh U Højsgaard S Yan J. The R Package geepack for generalized estimating equations. J Stat Softw. 2006;15:1–11.

[45] Greifer N. Cobalt: covariate balance tables and plots. 2023. https://CRAN.R-project.org/package=cobalt. Accessed June 7, 2024.

[46] Sjoberg DD Whiting K Curry M Lavery JA Larmarange J. Reproducible summary tables with the gtsummary package. R J. 2021;13:570–80.

[47] Brintz BJ Madaras-Kelly K Nevers M Echevarria KL Goetz MB Samore MH. Predicting antibiotic resistance in Enterobacterales to support optimal empiric treatment of urinary tract infections in outpatient veterans. Antimicrob Steward Healthc Epidemiol. 2024;4:e118. 39257425 10.1017/ash.2024.377 PMC11384162

[48] Cortes-Penfield NW Trautner BW Jump RLP. Urinary tract infection and asymptomatic bacteriuria in older adults. Infect Dis Clin North Am. 2017;31:673–88. 29079155 10.1016/j.idc.2017.07.002 PMC5802407

[49] Rastogi R Martinez KA Gupta N Rood M Rothberg MB. Management of urinary tract infections in direct to consumer telemedicine. J Gen Intern Med. 2020;35:643–8. 31667749 10.1007/s11606-019-05415-7 PMC7080949

[50] O’Shea AMJ Gibson M Merchant J. Understanding rural-urban differences in veterans’ internet access, use and patient preferences for telemedicine. J Rural Health. 2024;40:438–45. 37935649 10.1111/jrh.12805

