# Supplementary material for: The comparative effectiveness of telehealth versus primary care and collection of urine cultures on outcome in urinary tract infection
Source: Medicine (Baltimore). 2025 Jul 18;104(29):e43172. doi: 10.1097/MD.0000000000043172 (PMC12282790; doi:10.1097/MD.0000000000043172)
Supplement: Supplementary file 1 [file medi-104-e43172-s001.docx]

**Supplemental Material**

**Supplement 1. UTI-Related ICD-10 codes used to identify Index Visit**

| **ICD10Code** | **ICD10Description** |
| --- | --- |
| N10. | Acute pyelonephritis |
| N11.0 | Nonobstructive reflux-associated chronic pyelonephritis |
| N11.1 | Chronic obstructive pyelonephritis |
| N13.6 | Pyonephrosis |
| N30.00 | Acute cystitis without hematuria |
| N30.01 | Acute cystitis with hematuria |
| N30.20 | Other chronic cystitis without hematuria |
| N30.21 | Other chronic cystitis with hematuria |
| N30.80 | Other cystitis without hematuria |
| N30.81 | Other cystitis with hematuria |
| N30.90 | Cystitis, unspecified without hematuria |
| N30.91 | Cystitis, unspecified with hematuria |
| N39.0 | Urinary tract infection, site not specified |
| N41.0 | Acute prostatitis |
| R30.0 | Dysuria |
| R30.1 | Vesical tenesmus |
| R30.9 | Painful micturition, unspecified |
| R35.0 | Frequency of micturition |
| R39.11 | Hesitancy of micturition |
| R39.12 | Poor urinary stream |
| R39.13 | Splitting of urinary stream |
| R39.14 | Feeling of incomplete bladder emptying |
| R39.15 | Urgency of urination |
| R39.16 | Straining to void |
| R39.191 | Need to immediately re-void |
| R39.192 | Position dependent micturition |
| R39.198 | Other difficulties with micturition |
|  |  |

**Supplement 1 ICD-10/CPT codes used to identify urological procedure (exclusion criteria).**

- ICD-10 procedure codes: 0VT00ZZ, 0VT04ZZ, 0VT07ZZ, 0VT08ZZ (TURP), and 0T7D0DZ, 0T7D0ZZ, 0T7D3DZ, 0T7D3ZZ, 0T7D4DZ, 0T7D4ZZ, 0T7D7DZ, 0T7D7ZZ, 0T7D8DZ, 0T7D8ZZ (cysto).
- We also searched CPT names for the strings “cysto”, “turp”, and “prostat”.
- The table below shows the codes we found—i.e., the codes that caused visits to be excluded.

| **ProcedureCodingSystem** | **ProcedureCode** | **ProcedureDescription** |
| --- | --- | --- |
|  |  |  |
| CPT | 52332 | CYSTOURETHROSCOPY, WITH INSERTION OF INDWELLING URETERAL STENT (EG, GIBBONS OR DOUBLE-J TYPE) |
| CPT | 52000 | CYSTOURETHROSCOPY (SEPARATE PROCEDURE) |
| CPT | 52356 | CYSTOURETHROSCOPY, WITH URETEROSCOPY AND/OR PYELOSCOPY; WITH LITHOTRIPSY INCLUDING INSERTION OF INDWELLING URETERAL STENT (EG, GIBBONS OR DOUBLE-J TYPE) |
| CPT | 52310 | CYSTOURETHROSCOPY, WITH REMOVAL OF FOREIGN BODY, CALCULUS, OR URETERAL STENT FROM URETHRA OR BLADDER (SEPARATE PROCEDURE); SIMPLE |
| CPT | 52001 | CYSTOURETHROSCOPY WITH IRRIGATION AND EVACUATION OF MULTIPLE OBSTRUCTING CLOTS |
| CPT | 52351 | CYSTOURETHROSCOPY, WITH URETEROSCOPY AND/OR PYELOSCOPY; DIAGNOSTIC |
| CPT | 52281 | CYSTOURETHROSCOPY, WITH CALIBRATION AND/OR DILATION OF URETHRAL STRICTURE OR STENOSIS, WITH OR WITHOUT MEATOTOMY, WITH OR WITHOUT INJECTION PROCEDURE FOR CYSTOGRAPHY, MALE OR FEMALE |
| CPT | 52601 | TRANSURETHRAL ELECTROSURGICAL RESECTION OF PROSTATE, INCLUDING CONTROL OF POSTOPERATIVE BLEEDING, COMPLETE (VASECTOMY, MEATOTOMY, CYSTOURETHROSCOPY, URETHRAL CALIBRATION AND/OR DILATION, AND INTERNAL URETHROTOMY ARE INCLUDED) |
| CPT | 52352 | CYSTOURETHROSCOPY, WITH URETEROSCOPY AND/OR PYELOSCOPY; WITH REMOVAL OR MANIPULATION OF CALCULUS (URETERAL CATHETERIZATION IS INCLUDED) |
| CPT | 52315 | CYSTOURETHROSCOPY, WITH REMOVAL OF FOREIGN BODY, CALCULUS, OR URETERAL STENT FROM URETHRA OR BLADDER (SEPARATE PROCEDURE); COMPLICATED |
| CPT | 52204 | CYSTOURETHROSCOPY, WITH BIOPSY(S) |
| CPT | 52353 | CYSTOURETHROSCOPY, WITH URETEROSCOPY AND/OR PYELOSCOPY; WITH LITHOTRIPSY (URETERAL CATHETERIZATION IS INCLUDED) |
| CPT | 55700 | BIOPSY, PROSTATE; NEEDLE OR PUNCH, SINGLE OR MULTIPLE, ANY APPROACH |
| CPT | 52005 | CYSTOURETHROSCOPY, WITH URETERAL CATHETERIZATION, WITH OR WITHOUT IRRIGATION, INSTILLATION, OR URETEROPYELOGRAPHY, EXCLUSIVE OF RADIOLOGIC SERVICE; |
| CPT | 52214 | CYSTOURETHROSCOPY, WITH FULGURATION (INCLUDING CRYOSURGERY OR LASER SURGERY) OF TRIGONE, BLADDER NECK, PROSTATIC FOSSA, URETHRA, OR PERIURETHRAL GLANDS |
| ICD10 | 0T7D8ZZ | Dilation of Urethra, Via Natural or Artificial Opening Endoscopic |
| CPT | 52240 | CYSTOURETHROSCOPY, WITH FULGURATION (INCLUDING CRYOSURGERY OR LASER SURGERY) AND/OR RESECTION OF; LARGE BLADDER TUMOR(S) |
| CPT | 52224 | CYSTOURETHROSCOPY, WITH FULGURATION (INCLUDING CRYOSURGERY OR LASER SURGERY) OR TREATMENT OF MINOR (LESS THAN 0.5 CM) LESION(S) WITH OR WITHOUT BIOPSY |
| CPT | G0102 | PROSTATE CANCER SCREENING; DIGITAL RECTAL EXAMINATION |
| CPT | 00914 | ANESTHESIA FOR TRANSURETHRAL PROCEDURES (INCLUDING URETHROCYSTOSCOPY); TRANSURETHRAL RESECTION OF PROSTATE |
| CPT | C2627 | CATHETER, SUPRAPUBIC/CYSTOSCOPIC |
| CPT | 51728 | COMPLEX CYSTOMETROGRAM (IE, CALIBRATED ELECTRONIC EQUIPMENT); WITH VOIDING PRESSURE STUDIES (IE, BLADDER VOIDING PRESSURE), ANY TECHNIQUE |
| CPT | 52234 | CYSTOURETHROSCOPY, WITH FULGURATION (INCLUDING CRYOSURGERY OR LASER SURGERY) AND/OR RESECTION OF; SMALL BLADDER TUMOR(S) (0.5 UP TO 2.0 CM) |
| ICD10 | 0VT08ZZ | Resection of Prostate, Via Natural or Artificial Opening Endoscopic |
| CPT | 51725 | SIMPLE CYSTOMETROGRAM (CMG) (EG, SPINAL MANOMETER) |
| CPT | 52700 | TRANSURETHRAL DRAINAGE OF PROSTATIC ABSCESS |
| CPT | 52235 | CYSTOURETHROSCOPY, WITH FULGURATION (INCLUDING CRYOSURGERY OR LASER SURGERY) AND/OR RESECTION OF; MEDIUM BLADDER TUMOR(S) (2.0 TO 5.0 CM) |
| CPT | 51726 | COMPLEX CYSTOMETROGRAM (IE, CALIBRATED ELECTRONIC EQUIPMENT); |
| ICD10 | 0T7D8DZ | Dilation of Urethra with Intraluminal Device, Via Natural or Artificial Opening Endoscopic |
| CPT | 51729 | COMPLEX CYSTOMETROGRAM (IE, CALIBRATED ELECTRONIC EQUIPMENT); WITH VOIDING PRESSURE STUDIES (IE, BLADDER VOIDING PRESSURE) AND URETHRAL PRESSURE PROFILE STUDIES (IE, URETHRAL CLOSURE PRESSURE PROFILE), ANY TECHNIQUE |
| CPT | 52341 | CYSTOURETHROSCOPY; WITH TREATMENT OF URETERAL STRICTURE (EG, BALLOON DILATION, LASER, ELECTROCAUTERY, AND INCISION) |
| CPT | 52354 | CYSTOURETHROSCOPY, WITH URETEROSCOPY AND/OR PYELOSCOPY; WITH BIOPSY AND/OR FULGURATION OF URETERAL OR RENAL PELVIC LESION |
| CPT | 52441 | CYSTOURETHROSCOPY, WITH INSERTION OF PERMANENT ADJUSTABLE TRANSPROSTATIC IMPLANT; SINGLE IMPLANT |
| CPT | 52442 | CYSTOURETHROSCOPY, WITH INSERTION OF PERMANENT ADJUSTABLE TRANSPROSTATIC IMPLANT; EACH ADDITIONAL PERMANENT ADJUSTABLE TRANSPROSTATIC IMPLANT (LIST SEPARATELY IN ADDITION TO CODE FOR PRIMARY PROCEDURE) |
| CPT | 52630 | TRANSURETHRAL RESECTION; RESIDUAL OR REGROWTH OF OBSTRUCTIVE PROSTATE TISSUE INCLUDING CONTROL OF POSTOPERATIVE BLEEDING, COMPLETE (VASECTOMY, MEATOTOMY, CYSTOURETHROSCOPY, URETHRAL CALIBRATION AND/OR DILATION, AND INTERNAL URETHROTOMY ARE INCLUDED) |
| CPT | 00865 | ANESTHESIA FOR EXTRAPERITONEAL PROCEDURES IN LOWER ABDOMEN, INCLUDING URINARY TRACT; RADICAL PROSTATECTOMY (SUPRAPUBIC, RETROPUBIC) |
| CPT | 52260 | CYSTOURETHROSCOPY, WITH DILATION OF BLADDER FOR INTERSTITIAL CYSTITIS; GENERAL OR CONDUCTION (SPINAL) ANESTHESIA |
| CPT | 52344 | CYSTOURETHROSCOPY WITH URETEROSCOPY; WITH TREATMENT OF URETERAL STRICTURE (EG, BALLOON DILATION, LASER, ELECTROCAUTERY, AND INCISION) |
| CPT | 52648 | LASER VAPORIZATION OF PROSTATE, INCLUDING CONTROL OF POSTOPERATIVE BLEEDING, COMPLETE (VASECTOMY, MEATOTOMY, CYSTOURETHROSCOPY, URETHRAL CALIBRATION AND/OR DILATION, INTERNAL URETHROTOMY AND TRANSURETHRAL RESECTION OF PROSTATE ARE INCLUDED IF PERFORMED) |
| CPT | 55720 | PROSTATOTOMY, EXTERNAL DRAINAGE OF PROSTATIC ABSCESS, ANY APPROACH; SIMPLE |
| CPT | 52287 | CYSTOURETHROSCOPY, WITH INJECTION(S) FOR CHEMODENERVATION OF THE BLADDER |
| CPT | 52320 | CYSTOURETHROSCOPY (INCLUDING URETERAL CATHETERIZATION); WITH REMOVAL OF URETERAL CALCULUS |
| CPT | 51727 | COMPLEX CYSTOMETROGRAM (IE, CALIBRATED ELECTRONIC EQUIPMENT); WITH URETHRAL PRESSURE PROFILE STUDIES (IE, URETHRAL CLOSURE PRESSURE PROFILE), ANY TECHNIQUE |
| CPT | 52450 | TRANSURETHRAL INCISION OF PROSTATE |
| ICD10 | 0T7D7ZZ | Dilation of Urethra, Via Natural or Artificial Opening |
| CPT | 52276 | CYSTOURETHROSCOPY WITH DIRECT VISION INTERNAL URETHROTOMY |
| CPT | 52282 | CYSTOURETHROSCOPY, WITH INSERTION OF PERMANENT URETHRAL STENT |
| CPT | 52300 | CYSTOURETHROSCOPY; WITH RESECTION OR FULGURATION OF ORTHOTOPIC URETEROCELE(S), UNILATERAL OR BILATERAL |
| CPT | 52342 | CYSTOURETHROSCOPY; WITH TREATMENT OF URETEROPELVIC JUNCTION STRICTURE (EG, BALLOON DILATION, LASER, ELECTROCAUTERY, AND INCISION) |
| CPT | 55801 | PROSTATECTOMY, PERINEAL, SUBTOTAL (INCLUDING CONTROL OF POSTOPERATIVE BLEEDING, VASECTOMY, MEATOTOMY, URETHRAL CALIBRATION AND/OR DILATION, AND INTERNAL URETHROTOMY) |
| CPT | 55821 | PROSTATECTOMY (INCLUDING CONTROL OF POSTOPERATIVE BLEEDING, VASECTOMY, MEATOTOMY, URETHRAL CALIBRATION AND/OR DILATION, AND INTERNAL URETHROTOMY); SUPRAPUBIC, SUBTOTAL, 1 OR 2 STAGES |
| CPT | 84066 | PHOSPHATASE, ACID; PROSTATIC |
| CPT | S2070 | CYSTOURETHROSCOPY, WITH URETEROSCOPY AND/OR PYELOSCOPY; WITH ENDOSCOPIC LASER TREATMENT OF URETERAL CALCULI (INCLUDES URETERAL CATHETERIZATION) |
| ICD10 | 0T7D4DZ | Dilation of Urethra with Intraluminal Device, Percutaneous Endoscopic Approach |
| ICD10 | 0VT04ZZ | Resection of Prostate, Percutaneous Endoscopic Approach |

**Supplement 1: ICD-10 codes for co-diagnoses potentially requiring antibiotic treatment on day of index visit (exclusion criteria)**

| **Codiagnosis_code** | **Codiagnosis_desc** |
| --- | --- |
|  |  |
| R10.9 | Unspecified abdominal pain |
| R10.30 | Lower abdominal pain, unspecified |
| R19.7 | Diarrhea, unspecified |
| A41.9 | Sepsis, unspecified organism |
| R11.2 | Nausea with vomiting, unspecified |
| B96.20 | Unspecified Escherichia coli [E. coli] as the cause of diseases classified elsewhere |
| R11.0 | Nausea |
| J18.9 | Pneumonia, unspecified organism |
| R10.84 | Generalized abdominal pain |
| J44.1 | Chronic obstructive pulmonary disease with (acute) exacerbation |
| R10.10 | Upper abdominal pain, unspecified |
| J01.90 | Acute sinusitis, unspecified |
| B96.1 | Klebsiella pneumoniae [K. pneumoniae] as the cause of diseases classified elsewhere |
| A64. | Unspecified sexually transmitted disease |
| L03.90 | Cellulitis, unspecified |
| B35.6 | Tinea cruris |
| J32.9 | Chronic sinusitis, unspecified |
| J02.9 | Acute pharyngitis, unspecified |
| B35.1 | Tinea unguium |
| R10.31 | Right lower quadrant pain |
| B96.89 | Other specified bacterial agents as the cause of diseases classified elsewhere |
| R10.32 | Left lower quadrant pain |
| B95.2 | Enterococcus as the cause of diseases classified elsewhere |
| J15.9 | Unspecified bacterial pneumonia |
| A41.51 | Sepsis due to Escherichia coli [E. coli] |
| L03.116 | Cellulitis of left lower limb |
| A41.89 | Other specified sepsis |
| R10.13 | Epigastric pain |
| L03.115 | Cellulitis of right lower limb |
| A09. | Infectious gastroenteritis and colitis, unspecified |
| R10.11 | Right upper quadrant pain |
| B35.3 | Tinea pedis |
| B96.5 | Pseudomonas (aeruginosa) (mallei) (pseudomallei) as the cause of diseases classified elsewhere |
| B96.4 | Proteus (mirabilis) (morganii) as the cause of diseases classified elsewhere |
| B35.4 | Tinea corporis |
| J01.80 | Other acute sinusitis |
| J01.00 | Acute maxillary sinusitis, unspecified |
| J18.8 | Other pneumonia, unspecified organism |
| A41.50 | Gram-negative sepsis, unspecified |
| L08.9 | Local infection of the skin and subcutaneous tissue, unspecified |
| J44.0 | Chronic obstructive pulmonary disease with (acute) lower respiratory infection |
| B96.29 | Other Escherichia coli [E. coli] as the cause of diseases classified elsewhere |
| R15.9 | Full incontinence of feces |
| R13.12 | Dysphagia, oropharyngeal phase |
| R14.0 | Abdominal distension (gaseous) |
| R13.19 | Other dysphagia |
| R19.4 | Change in bowel habit |
| J18.1 | Lobar pneumonia, unspecified organism |
| A04.72 | Enterocolitis due to Clostridium difficile, not specified as recurrent |
| J32.8 | Other chronic sinusitis |
| A41.59 | Other Gram-negative sepsis |
| R12. | Heartburn |
| L70.9 | Acne, unspecified |
| H66.91 | Otitis media, unspecified, right ear |
| J01.10 | Acute frontal sinusitis, unspecified |
| R14.3 | Flatulence |
| J17. | Pneumonia in diseases classified elsewhere |
| J18.0 | Bronchopneumonia, unspecified organism |
| R16.0 | Hepatomegaly, not elsewhere classified |
| J32.0 | Chronic maxillary sinusitis |
| L03.119 | Cellulitis of unspecified part of limb |
| A49.9 | Bacterial infection, unspecified |
| B95.61 | Methicillin susceptible Staphylococcus aureus infection as the cause of diseases classified elsewhere |
| R19.09 | Other intra-abdominal and pelvic swelling, mass and lump |
| A08.39 | Other viral enteritis |
| A59.01 | Trichomonal vulvovaginitis |
| L03.818 | Cellulitis of other sites |
| R10.12 | Left upper quadrant pain |
| H66.92 | Otitis media, unspecified, left ear |
| L02.91 | Cutaneous abscess, unspecified |
| A41.81 | Sepsis due to Enterococcus |
| A49.02 | Methicillin resistant Staphylococcus aureus infection, unspecified site |
| A63.8 | Other specified predominantly sexually transmitted diseases |
| B95.1 | Streptococcus, group B, as the cause of diseases classified elsewhere |
| B95.7 | Other staphylococcus as the cause of diseases classified elsewhere |
| L03.031 | Cellulitis of right toe |
| A59.9 | Trichomoniasis, unspecified |
| R16.1 | Splenomegaly, not elsewhere classified |
| B35.9 | Dermatophytosis, unspecified |
| B95.62 | Methicillin resistant Staphylococcus aureus infection as the cause of diseases classified elsewhere |
| A54.9 | Gonococcal infection, unspecified |
| B95.4 | Other streptococcus as the cause of diseases classified elsewhere |
| R10.814 | Left lower quadrant abdominal tenderness |
| R16.2 | Hepatomegaly with splenomegaly, not elsewhere classified |
| J02.0 | Streptococcal pharyngitis |
| L03.011 | Cellulitis of right finger |
| L70.8 | Other acne |
| R10.819 | Abdominal tenderness, unspecified site |
| B96.81 | Helicobacter pylori [H. pylori] as the cause of diseases classified elsewhere |
| J32.1 | Chronic frontal sinusitis |
| L03.032 | Cellulitis of left toe |
| L03.311 | Cellulitis of abdominal wall |
| L70.0 | Acne vulgaris |
| A53.9 | Syphilis, unspecified |
| J16.8 | Pneumonia due to other specified infectious organisms |
| L03.114 | Cellulitis of left upper limb |
| R10.0 | Acute abdomen |
| R14.2 | Eructation |
| R19.00 | Intra-abdominal and pelvic swelling, mass and lump, unspecified site |
| A08.4 | Viral intestinal infection, unspecified |
| A41.52 | Sepsis due to Pseudomonas |
| A56.01 | Chlamydial cystitis and urethritis |
| A59.03 | Trichomonal cystitis and urethritis |
| H66.93 | Otitis media, unspecified, bilateral |
| L03.315 | Cellulitis of perineum |
| R15.1 | Fecal smearing |
| R15.2 | Fecal urgency |
| R19.8 | Other specified symptoms and signs involving the digestive system and abdomen |
| A66.2 | Other early skin lesions of yaws |
| A74.9 | Chlamydial infection, unspecified |
| B86. | Scabies |
| J01.91 | Acute recurrent sinusitis, unspecified |
| L03.211 | Cellulitis of face |
| A49.8 | Other bacterial infections of unspecified site |
| B95.8 | Unspecified staphylococcus as the cause of diseases classified elsewhere |
| H66.90 | Otitis media, unspecified, unspecified ear |
| J01.81 | Other acute recurrent sinusitis |
| J03.90 | Acute tonsillitis, unspecified |
| L02.212 | Cutaneous abscess of back [any part, except buttock] |
| L02.224 | Furuncle of groin |
| L02.818 | Cutaneous abscess of other sites |
| L03.012 | Cellulitis of left finger |
| L03.317 | Cellulitis of buttock |
| L08.89 | Other specified local infections of the skin and subcutaneous tissue |
| N15.1 | Renal and perinephric abscess |
| R10.33 | Periumbilical pain |
| R10.813 | Right lower quadrant abdominal tenderness |
| R10.816 | Epigastric abdominal tenderness |
| R13.14 | Dysphagia, pharyngoesophageal phase |
| R19.07 | Generalized intra-abdominal and pelvic swelling, mass and lump |
| B36.0 | Pityriasis versicolor |
| J01.01 | Acute recurrent maxillary sinusitis |
| J01.11 | Acute recurrent frontal sinusitis |
| L01.00 | Impetigo, unspecified |
| L03.019 | Cellulitis of unspecified finger |
| L03.312 | Cellulitis of back [any part except buttock] |
| L04.0 | Acute lymphadenitis of face, head and neck |
| R10.811 | Right upper quadrant abdominal tenderness |
| R13.11 | Dysphagia, oral phase |
| R13.13 | Dysphagia, pharyngeal phase |
| A41.01 | Sepsis due to Methicillin susceptible Staphylococcus aureus |
| A59.00 | Urogenital trichomoniasis, unspecified |
| B36.9 | Superficial mycosis, unspecified |
| H66.004 | Acute suppurative otitis media without spontaneous rupture of ear drum, recurrent, right ear |
| K12.2 | Cellulitis and abscess of mouth |
| K12.30 | Oral mucositis (ulcerative), unspecified |
| L02.213 | Cutaneous abscess of chest wall |
| L02.215 | Cutaneous abscess of perineum |
| L02.31 | Cutaneous abscess of buttock |
| L02.32 | Furuncle of buttock |
| L02.92 | Furuncle, unspecified |
| L03.039 | Cellulitis of unspecified toe |
| L03.113 | Cellulitis of right upper limb |
| L03.319 | Cellulitis of trunk, unspecified |
| L05.91 | Pilonidal cyst without abscess |
| A56.8 | Sexually transmitted chlamydial infection of other sites |
| B95.5 | Unspecified streptococcus as the cause of diseases classified elsewhere |
| B99.9 | Unspecified infectious disease |
| H66.002 | Acute suppurative otitis media without spontaneous rupture of ear drum, left ear |
| H67.1 | Otitis media in diseases classified elsewhere, right ear |
| H70.11 | Chronic mastoiditis, right ear |
| J13. | Pneumonia due to Streptococcus pneumoniae |
| J15.0 | Pneumonia due to Klebsiella pneumoniae |
| L02.214 | Cutaneous abscess of groin |
| L02.414 | Cutaneous abscess of left upper limb |
| L03.314 | Cellulitis of groin |
| L04.9 | Acute lymphadenitis, unspecified |
| L73.1 | Pseudofolliculitis barbae |
| L73.8 | Other specified follicular disorders |
| R14.1 | Gas pain |
| A06.81 | Amebic cystitis |
| A06.82 | Other amebic genitourinary infections |
| A07.0 | Balantidiasis |
| A31.0 | Pulmonary mycobacterial infection |
| A46. | Erysipelas |
| A54.00 | Gonococcal infection of lower genitourinary tract, unspecified |
| A54.01 | Gonococcal cystitis and urethritis, unspecified |
| A56.19 | Other chlamydial genitourinary infection |
| A56.2 | Chlamydial infection of genitourinary tract, unspecified |
| A59.09 | Other urogenital trichomoniasis |
| J02.8 | Acute pharyngitis due to other specified organisms |
| L02.211 | Cutaneous abscess of abdominal wall |
| L02.234 | Carbuncle of groin |
| L02.415 | Cutaneous abscess of right lower limb |
| L02.416 | Cutaneous abscess of left lower limb |
| L02.511 | Cutaneous abscess of right hand |
| L03.313 | Cellulitis of chest wall |
| L73.0 | Acne keloid |
| R10.812 | Left upper quadrant abdominal tenderness |
| R10.817 | Generalized abdominal tenderness |
| R18.0 | Malignant ascites |
| A02.0 | Salmonella enteritis |
| A04.4 | Other intestinal Escherichia coli infections |
| A04.5 | Campylobacter enteritis |
| A15.0 | Tuberculosis of lung |
| A41.02 | Sepsis due to Methicillin resistant Staphylococcus aureus |
| A41.1 | Sepsis due to other specified staphylococcus |
| A41.2 | Sepsis due to unspecified staphylococcus |
| A49.1 | Streptococcal infection, unspecified site |
| A49.2 | Hemophilus influenzae infection, unspecified site |
| A51.31 | Condyloma latum |
| A52.17 | General paresis |
| A53.0 | Latent syphilis, unspecified as early or late |
| A54.03 | Gonococcal cervicitis, unspecified |
| A54.23 | Gonococcal infection of other male genital organs |
| A57. | Chancroid |
| A66.9 | Yaws, unspecified |
| A68.9 | Relapsing fever, unspecified |
| A69.20 | Lyme disease, unspecified |
| B35.8 | Other dermatophytoses |
| B47.9 | Mycetoma, unspecified |
| B85.2 | Pediculosis, unspecified |
| B88.9 | Infestation, unspecified |
| H66.014 | Acute suppurative otitis media with spontaneous rupture of ear drum, recurrent, right ear |
| H67.2 | Otitis media in diseases classified elsewhere, left ear |
| H67.3 | Otitis media in diseases classified elsewhere, bilateral |
| H70.90 | Unspecified mastoiditis, unspecified ear |
| J01.20 | Acute ethmoidal sinusitis, unspecified |
| J01.30 | Acute sphenoidal sinusitis, unspecified |
| J15.8 | Pneumonia due to other specified bacteria |
| J32.4 | Chronic pansinusitis |
| L02.01 | Cutaneous abscess of face |
| L02.11 | Cutaneous abscess of neck |
| L02.222 | Furuncle of back [any part, except buttock] |
| L02.223 | Furuncle of chest wall |
| L02.231 | Carbuncle of abdominal wall |
| L02.33 | Carbuncle of buttock |
| L02.413 | Cutaneous abscess of right upper limb |
| L02.422 | Furuncle of left axilla |
| L03.811 | Cellulitis of head [any part, except face] |
| L05.01 | Pilonidal cyst with abscess |
| L05.92 | Pilonidal sinus without abscess |
| L88. | Pyoderma gangrenosum |
| M72.6 | Necrotizing fasciitis |
| R10.823 | Right lower quadrant rebound abdominal tenderness |
| R10.83 | Colic |
| R19.01 | Right upper quadrant abdominal swelling, mass and lump |
| R19.02 | Left upper quadrant abdominal swelling, mass and lump |
| R19.03 | Right lower quadrant abdominal swelling, mass and lump |
| R19.04 | Left lower quadrant abdominal swelling, mass and lump |
| A02.29 | Salmonella with other localized infection |
| A04.0 | Enteropathogenic Escherichia coli infection |
| A04.2 | Enteroinvasive Escherichia coli infection |
| A04.9 | Bacterial intestinal infection, unspecified |
| A05.8 | Other specified bacterial foodborne intoxications |
| A08.19 | Acute gastroenteropathy due to other small round viruses |
| A08.2 | Adenoviral enteritis |
| A18.2 | Tuberculous peripheral lymphadenopathy |
| A18.32 | Tuberculous enteritis |
| A31.2 | Disseminated mycobacterium avium-intracellulare complex (DMAC) |
| A31.9 | Mycobacterial infection, unspecified |
| A35. | Other tetanus |
| A40.1 | Sepsis due to streptococcus, group B |
| A40.3 | Sepsis due to Streptococcus pneumoniae |
| A41.53 | Sepsis due to Serratia |
| A48.8 | Other specified bacterial diseases |
| A49.01 | Methicillin susceptible Staphylococcus aureus infection, unspecified site |
| A49.3 | Mycoplasma infection, unspecified site |
| A52.10 | Symptomatic neurosyphilis, unspecified |
| A54.09 | Other gonococcal infection of lower genitourinary tract |
| A54.24 | Gonococcal female pelvic inflammatory disease |
| A54.6 | Gonococcal infection of anus and rectum |
| A56.00 | Chlamydial infection of lower genitourinary tract, unspecified |
| A56.02 | Chlamydial vulvovaginitis |
| A56.09 | Other chlamydial infection of lower genitourinary tract |
| A56.3 | Chlamydial infection of anus and rectum |
| A59.02 | Trichomonal prostatitis |
| A74.89 | Other chlamydial diseases |
| B35.0 | Tinea barbae and tinea capitis |
| B36.8 | Other specified superficial mycoses |
| B88.2 | Other arthropod infestations |
| B88.8 | Other specified infestations |
| B89. | Unspecified parasitic disease |
| B95.0 | Streptococcus, group A, as the cause of diseases classified elsewhere |
| B95.3 | Streptococcus pneumoniae as the cause of diseases classified elsewhere |
| B96.0 | Mycoplasma pneumoniae [M. pneumoniae] as the cause of diseases classified elsewhere |
| B96.6 | Bacteroides fragilis [B. fragilis] as the cause of diseases classified elsewhere |
| B96.7 | Clostridium perfringens [C. perfringens] as the cause of diseases classified elsewhere |
| B99.8 | Other infectious disease |
| G03.9 | Meningitis, unspecified |
| G04.01 | Postinfectious acute disseminated encephalitis and encephalomyelitis (postinfectious ADEM) |
| G04.31 | Postinfectious acute necrotizing hemorrhagic encephalopathy |
| G04.89 | Other myelitis |
| G14. | Postpolio syndrome |
| H66.001 | Acute suppurative otitis media without spontaneous rupture of ear drum, right ear |
| H66.012 | Acute suppurative otitis media with spontaneous rupture of ear drum, left ear |
| H66.3X1 | Other chronic suppurative otitis media, right ear |
| H66.40 | Suppurative otitis media, unspecified, unspecified ear |
| H66.41 | Suppurative otitis media, unspecified, right ear |
| H70.003 | Acute mastoiditis without complications, bilateral |
| H70.10 | Chronic mastoiditis, unspecified ear |
| H70.893 | Other mastoiditis and related conditions, bilateral |
| H95.192 | Other disorders following mastoidectomy, left ear |
| J01.40 | Acute pansinusitis, unspecified |
| J03.00 | Acute streptococcal tonsillitis, unspecified |
| J03.91 | Acute recurrent tonsillitis, unspecified |
| J15.4 | Pneumonia due to other streptococci |
| J15.6 | Pneumonia due to other Gram-negative bacteria |
| J15.7 | Pneumonia due to Mycoplasma pneumoniae |
| J32.2 | Chronic ethmoidal sinusitis |
| J32.3 | Chronic sphenoidal sinusitis |
| K90.81 | Whipple's disease |
| L01.01 | Non-bullous impetigo |
| L01.02 | Bockhart's impetigo |
| L02.12 | Furuncle of neck |
| L02.219 | Cutaneous abscess of trunk, unspecified |
| L02.232 | Carbuncle of back [any part, except buttock] |
| L02.411 | Cutaneous abscess of right axilla |
| L02.412 | Cutaneous abscess of left axilla |
| L02.421 | Furuncle of right axilla |
| L02.429 | Furuncle of limb, unspecified |
| L02.436 | Carbuncle of left lower limb |
| L02.522 | Furuncle left hand |
| L02.631 | Carbuncle of right foot |
| L02.811 | Cutaneous abscess of head [any part, except face] |
| L02.821 | Furuncle of head [any part, except face] |
| L03.111 | Cellulitis of right axilla |
| L03.112 | Cellulitis of left axilla |
| L03.123 | Acute lymphangitis of right upper limb |
| L03.125 | Acute lymphangitis of right lower limb |
| L03.221 | Cellulitis of neck |
| L04.1 | Acute lymphadenitis of trunk |
| L04.8 | Acute lymphadenitis of other sites |
| L08.82 | Omphalitis not of newborn |
| L92.8 | Other granulomatous disorders of the skin and subcutaneous tissue |
| L98.0 | Pyogenic granuloma |
| R11.12 | Projectile vomiting |
| R13.0 | Aphagia |
| R15.0 | Incomplete defecation |
| R19.15 | Other abnormal bowel sounds |

**Supplement 2: Clinic Stop Codes used to Define Telehealth and Primary Care Visits**

Telehealth visits were identified by review of primary and/or secondary stop codes for encounters with the terms “telehealth”, “telephone”, “video”, “vid”, or “secure messaging” in which all inclusion and no exclusion criteria were met.

| **Telehealth (n=16,697)** |  |  |
| --- | --- | --- |
| **PrimaryStopName** | **SecondaryStopName** | **n= visits** |
| TELEPHONE PRIMARY CARE | PRIMARY CARE/MEDICINE | 4040 |
| TELEPHONE PRIMARY CARE | *Unknown at this time* | 2403 |
| TELEPHONE PRIMARY CARE | NURSING (2ND ONLY) | 2108 |
| TELEPHONE PRIMARY CARE | NURSE PRACTITIONER | 1408 |
| PRIMARY CARE/MEDICINE | RT CLIN VID CARE HOME | 1371 |
| TELEPHONE TRIAGE | TELEPHONE TRIAGE IN VISN | 1141 |
| TELEPHONE PRIMARY CARE | COMP WOMEN'S HLTH | 893 |
| PRIMARY CARE/MEDICINE | MHV SECURE MESSAGING | 410 |
| TELEPHONE PRIMARY CARE | PHYSICIAN ASSISTANT | 356 |
| TELEPHONE/MEDICINE | URGENT CARE CLINIC | 233 |
| COMP WOMEN'S HLTH | RT CLIN VID CARE HOME | 231 |
| PRIMARY CARE/MEDICINE | RT CLIN VID TH PAT SITE | 182 |
| TELEPHONE/SURGERY | GYNECOLOGY | 161 |
| TELEPHONE/GERIATRICS | GERIPACT | 149 |
| TELEPHONE TRIAGE | PRIMARY CARE/MEDICINE | 115 |
| TELEPHONE PRIMARY CARE | FELLOW/RESIDENT | 112 |
| URGENT CARE CLINIC | RT CLIN VID CARE HOME | 112 |
| TELEPHONE TRIAGE | *Unknown at this time* | 102 |
| TELEPHONE TRIAGE | URGENT CARE CLINIC | 88 |
| TELEPHONE TRIAGE | NURSING (2ND ONLY) | 80 |
| GENERAL INTERNAL MEDICINE | RT CLIN VID CARE HOME | 76 |
| TELEPHONE SCI | PRIMARY CARE/MEDICINE | 58 |
| TELEPHONE/SURGERY | *Unknown at this time* | 57 |
| SCI TELEHEALTH VIRTUAL | RT CLIN VID CARE HOME | 54 |
| COMP WOMEN'S HLTH | MHV SECURE MESSAGING | 53 |
| TELEPHONE PRIMARY CARE | GERIPACT | 48 |
| TELEPHONE PRIMARY CARE | GENERAL INTERNAL MEDICINE | 44 |
| TELEPHONE TRIAGE | NURSE PRACTITIONER | 40 |
| TELEPHONE/GERIATRICS | *Unknown at this time* | 39 |
| TELEPHONE/MEDICINE | *Unknown at this time* | 35 |
| TELEPHONE SCI | *Unknown at this time* | 34 |
| TELEPHONE SCI | NURSING (2ND ONLY) | 31 |
| GERIPACT | RT CLIN VID CARE HOME | 28 |
| TELEPHONE SCI | NURSE PRACTITIONER | 26 |
| GYNECOLOGY | RT CLIN VID CARE HOME | 25 |
| TELEPHONE/MEDICINE | GENERAL INTERNAL MEDICINE | 23 |
| TELEPHONE/GERIATRICS | NURSE PRACTITIONER | 20 |
| TELEPHONE/MEDICINE | GYNECOLOGY | 20 |
| TELEPHONE/SURGERY | NURSING (2ND ONLY) | 19 |
| TELEPHONE/GERIATRICS | GERI PROB CONSULT CLINIC | 18 |
| TELEPHONE/MEDICINE | NURSE PRACTITIONER | 16 |
| TELEPHONE/MEDICINE | PRIMARY CARE/MEDICINE | 16 |
| TELEPHONE/GERIATRICS | NURSING (2ND ONLY) | 12 |
| TELEPHONE/MEDICINE | NURSING (2ND ONLY) | 12 |
| 52 Additional clinic descriptors | (< 10 visits/descriptor) | 158 |
|  |  |  |
| **Primary Care (n=21,297)** |  |  |
| **PrimaryStopName** | **SecondaryStopName** | **n= visits** |
| PRIMARY CARE/MEDICINE | *Unknown at this time* | 9183 |
| PRIMARY CARE/MEDICINE | NURSE PRACTITIONER | 5667 |
| URGENT CARE CLINIC | *Unknown at this time* | 4222 |
| PRIMARY CARE/MEDICINE | NURSING (2ND ONLY) | 3475 |
| PRIMARY CARE/MEDICINE | PHYSICIAN ASSISTANT | 1273 |
| PRIMARY CARE/MEDICINE | FELLOW/RESIDENT | 1031 |
| GENERAL INTERNAL MEDICINE | *Unknown at this time* | 987 |
| COMP WOMEN'S HLTH | NURSE PRACTITIONER | 872 |
| COMP WOMEN'S HLTH | *Unknown at this time* | 828 |
| GENERAL INTERNAL MEDICINE | NURSE PRACTITIONER | 438 |
| GENERAL INTERNAL MEDICINE | PRIMARY CARE/MEDICINE | 422 |
| COMP WOMEN'S HLTH | NURSING (2ND ONLY) | 416 |
| GENERAL INTERNAL MEDICINE | NURSING (2ND ONLY) | 292 |
| GERIPACT | *Unknown at this time* | 212 |
| GYNECOLOGY | *Unknown at this time* | 181 |
| COMP WOMEN'S HLTH | PRIMARY CARE/MEDICINE | 166 |
| URGENT CARE CLINIC | PRIMARY CARE/MEDICINE | 134 |
| GERIPACT | NURSE PRACTITIONER | 97 |
| COMP WOMEN'S HLTH | PHYSICIAN ASSISTANT | 79 |
| COMP WOMEN'S HLTH | FELLOW/RESIDENT | 73 |
| GERIPACT | NURSING (2ND ONLY) | 59 |
| GYNECOLOGY | NURSE PRACTITIONER | 56 |
| GENERAL INTERNAL MEDICINE | PHYSICIAN ASSISTANT | 47 |
| PRIMARY CARE/MEDICINE | CLINICAL NURSE SPECIALIST | 30 |
| COMP WOMEN'S HLTH | GYNECOLOGY | 26 |
| URGENT CARE CLINIC | CHART CONSULT | 20 |
| PRIMARY CARE/MEDICINE | CHART CONSULT | 19 |
| PRIMARY CARE/MEDICINE | COMP WOMEN'S HLTH | 18 |
| GYNECOLOGY | COMP WOMEN'S HLTH | 15 |
| GYNECOLOGY | PHYSICIAN ASSISTANT | 15 |
| PRIMARY CARE/MEDICINE | RT CLIN VD TH PRV SITE(DIFSTA) | 15 |
| GENERAL INTERNAL MEDICINE | CHART CONSULT | 13 |
| GERIPACT | FELLOW/RESIDENT | 13 |
| GENERAL INTERNAL MEDICINE | FELLOW/RESIDENT | 12 |
| GERI PROB CONSULT CLINIC | *Unknown at this time* | 12 |
| 26 Additional clinic descriptors | (< 10 visits/descriptor) | 62 |

**Supplement 3. ICD-10 Codes Used to Define Clinical Failure.**

| ICD10Code | ICD10Description |
| --- | --- |
| A40.1 | Sepsis due to streptococcus, group B |
| A40.8 | Other streptococcal sepsis |
| A40.9 | Streptococcal sepsis, unspecified |
| A41.01 | Sepsis due to Methicillin susceptible Staphylococcus aureus |
| A41.02 | Sepsis due to Methicillin resistant Staphylococcus aureus |
| A41.1 | Sepsis due to other specified staphylococcus |
| A41.2 | Sepsis due to unspecified staphylococcus |
| A41.50 | Gram-negative sepsis, unspecified |
| A41.51 | Sepsis due to Escherichia coli [E. coli] |
| A41.52 | Sepsis due to Pseudomonas |
| A41.53 | Sepsis due to Serratia |
| A41.59 | Other Gram-negative sepsis |
| A41.81 | Sepsis due to Enterococcus |
| A41.89 | Other specified sepsis |
| A41.9 | Sepsis, unspecified organism |
| A54.86 | Gonococcal sepsis |
| A60.00 | Herpesviral infection of urogenital system, unspecified |
| B37.41 | Candidal cystitis and urethritis |
| B37.49 | Other urogenital candidiasis |
| C61. | Malignant neoplasm of prostate |
| C67.0 | Malignant neoplasm of trigone of bladder |
| C67.9 | Malignant neoplasm of bladder, unspecified |
| D07.5 | Carcinoma in situ of prostate |
| D29.1 | Benign neoplasm of prostate |
| D39.10 | Neoplasm of uncertain behavior of unspecified ovary |
| D49.4 | Neoplasm of unspecified behavior of bladder |
| D72.829 | Elevated white blood cell count, unspecified |
| K65.1 | Peritoneal abscess |
| N02.8 | Recurrent and persistent hematuria with other morphologic changes |
| N10. | Acute pyelonephritis |
| N12. | Tubulo-interstitial nephritis, not specified as acute or chronic |
| N13.30 | Unspecified hydronephrosis |
| N13.39 | Other hydronephrosis |
| N13.5 | Crossing vessel and stricture of ureter without hydronephrosis |
| N13.6 | Pyonephrosis |
| N13.8 | Other obstructive and reflux uropathy |
| N13.9 | Obstructive and reflux uropathy, unspecified |
| N17.9 | Acute kidney failure, unspecified |
| N20.0 | Calculus of kidney |
| N20.1 | Calculus of ureter |
| N20.2 | Calculus of kidney with calculus of ureter |
| N21.0 | Calculus in bladder |
| N21.1 | Calculus in urethra |
| N28.1 | Cyst of kidney, acquired |
| N28.89 | Other specified disorders of kidney and ureter |
| N30.00 | Acute cystitis without hematuria |
| N30.01 | Acute cystitis with hematuria |
| N30.10 | Interstitial cystitis (chronic) without hematuria |
| N30.20 | Other chronic cystitis without hematuria |
| N30.41 | Irradiation cystitis with hematuria |
| N30.80 | Other cystitis without hematuria |
| N30.90 | Cystitis, unspecified without hematuria |
| N31.2 | Flaccid neuropathic bladder, not elsewhere classified |
| N31.9 | Neuromuscular dysfunction of bladder, unspecified |
| N32.3 | Diverticulum of bladder |
| N32.89 | Other specified disorders of bladder |
| N35.814 | Other anterior urethral stricture, male |
| N35.919 | Unspecified urethral stricture, male, unspecified site |
| N39.0 | Urinary tract infection, site not specified |
| N39.41 | Urge incontinence |
| N39.43 | Post-void dribbling |
| N39.9 | Disorder of urinary system, unspecified |
| N40.0 | Benign prostatic hyperplasia without lower urinary tract symptoms |
| N40.1 | Benign prostatic hyperplasia with lower urinary tract symptoms |
| N41.0 | Acute prostatitis |
| N41.1 | Chronic prostatitis |
| N41.2 | Abscess of prostate |
| N41.9 | Inflammatory disease of prostate, unspecified |
| N45.1 | Epididymitis |
| N45.3 | Epididymo-orchitis |
| N50.811 | Right testicular pain |
| N50.82 | Scrotal pain |
| N76.0 | Acute vaginitis |
| N83.209 | Unspecified ovarian cyst, unspecified side |
| N89.9 | Noninflammatory disorder of vagina, unspecified |
| N93.9 | Abnormal uterine and vaginal bleeding, unspecified |
| Q54.1 | Hypospadias, penile |
| R30.0 | Dysuria |
| R30.9 | Painful micturition, unspecified |
| R31.0 | Gross hematuria |
| R31.9 | Hematuria, unspecified |
| R32. | Unspecified urinary incontinence |
| R33.8 | Other retention of urine |
| R33.9 | Retention of urine, unspecified |
| R35.0 | Frequency of micturition |
| R35.1 | Nocturia |
| R39.11 | Hesitancy of micturition |
| R39.15 | Urgency of urination |
| R39.81 | Functional urinary incontinence |
| R50.9 | Fever, unspecified |
| R65.21 | Severe sepsis with septic shock |
| R78.81 | Bacteremia |
| R82.81 | Pyuria |
| R82.998 | Other abnormal findings in urine |
| R93.422 | Abnormal radiologic findings on diagnostic imaging of left kidney |
| R97.20 | Elevated prostate specific antigen [PSA] |
| T83.021A | Displacement of indwelling urethral catheter, initial encounter |
| T83.038D | Leakage of other urinary catheter, subsequent encounter |
| T83.091A | Other mechanical complication of indwelling urethral catheter, initial encounter |
| T83.098A | Other mechanical complication of other urinary catheter, initial encounter |
| T83.511A | Infection and inflammatory reaction due to indwelling urethral catheter, initial encounter |
| T83.512A | Infection and inflammatory reaction due to nephrostomy catheter, initial encounter |
| T83.518A | Infection and inflammatory reaction due to other urinary catheter, initial encounter |
| Y73.1 | Therapeutic (nonsurgical) and rehabilitative gastroenterology and urology devices associated with adverse incidents |
| Y84.6 | Urinary catheterization as the cause of abnormal reaction of the patient, or of later complication, without mention of misadventure at the time of the procedure |
| Z16.12 | Extended spectrum beta lactamase (ESBL) resistance |
| Z16.35 | Resistance to multiple antimicrobial drugs |
| Z46.6 | Encounter for fitting and adjustment of urinary device |
| Z80.42 | Family history of malignant neoplasm of prostate |
| Z85.46 | Personal history of malignant neoplasm of prostate |
| Z85.51 | Personal history of malignant neoplasm of bladder |
| Z87.440 | Personal history of urinary (tract) infections |
| Z87.442 | Personal history of urinary calculi |
| Z90.6 | Acquired absence of other parts of urinary tract |
| Z93.6 | Other artificial openings of urinary tract status |

**Supplement 3:** Antibiotics administered to hospitalized clinical failures

| **DrugNameWithoutDose** | **n** |
| --- | --- |
| CEFTRIAXONE | 122 |
| PIPERACILLIN/TAZOBACTAM | 56 |
| CEFEPIME | 32 |
| VANCOMYCIN | 27 |
| AMOXICILLIN/CLAVULANATE | 25 |
| SULFAMETHOXAZOLE/TRIMETHOPRIM | 23 |
| AZITHROMYCIN | 21 |
| CIPROFLOXACIN | 19 |
| METRONIDAZOLE | 16 |
| NITROFURANTOIN | 15 |
| CEPHALEXIN | 14 |
| LEVOFLOXACIN | 14 |
| ERTAPENEM | 13 |
| CEFAZOLIN | 10 |
| MEROPENEM | 10 |
| CIPROFLOXACIN/DEXTROSE | 7 |
| AMOXICILLIN | 6 |
| CEFDINIR | 6 |
| CEFPODOXIME PROXETIL | 6 |
| DOXYCYCLINE | 6 |
| AMPICILLIN/SULBACTAM | 4 |
| AZTREONAM | 4 |
| CLINDAMYCIN | 3 |
| CEFAZOLIN/DEXTROSE | 2 |
| CEFUROXIME | 2 |
| FOSFOMYCIN | 2 |
| CEFOTETAN | 1 |
| CEFOXITIN | 1 |
| CEFTAZIDIME | 1 |
| DAPTOMYCIN | 1 |
| LINEZOLID | 1 |
